# Digital cytometry of leukocyte landscapes reveals shared correlates of rejection across organ transplants

**DOI:** 10.1172/JCI208686

**Published:** 2026-09-22

**Authors:** Patrick T. Gauthier, Louisa M.S. Gerhardt, Lorenz Jahn, Christian Hinze, Philip F. Halloran, INTERCOMEX, INTERHEART, INTERLUNG, and INTERLIVER investigators

**Affiliations:** 1 Alberta Transplant Applied Genomics Centre, Edmonton, Alberta, Canada.; 2 Transcriptome Sciences Inc., Edmonton, Alberta, Canada.; 3 Department of Medicine V, University Medical Centre Mannheim, University of Heidelberg, Mannheim, Germany.; 4 Department of Nephrology and Hypertension, Hannover Medical School, Hannover, Germany.; 5 University of Alberta, Edmonton, Alberta, Canada.; 6 See Supplemental Table 7 for affiliations.

**Keywords:** Clinical Research, Immunology, Transcriptomics, Transplantation

## Abstract

**BACKGROUND:**

Improved understanding of the cellular mechanisms of organ transplant rejection is needed for developing targeted therapies to improve outcomes. We used digital cytometry to estimate the digital leukocyte entities (DLE) shared by rejection in biopsies of kidney, heart, lung, and liver organ transplants.

**METHODS:**

We used CIBERSORTx with the LM22 leukocyte reference to estimate associations between 22 DLEs with rejection in 8,422 transplant biopsies — 4,898 kidney, 2,361 heart, 652 lung, and 538 liver.

**RESULTS:**

In all organs, rejection correlated with an increase in DLEs designated as classically activated macrophages and a decrease in DLEs designated as regulatory T cells. T cell–mediated rejection (TCMR) was associated with a massive increase in otherwise rare T cell DLEs designated as memory-activated CD4 and Tγδ, as well as follicular-helper CD4 and CD8. TCMR also had increased DLE-designated plasma cells, compatible with a role for the B cell lineage in antigen presentation in TCMR. Antibody-mediated rejection (ABMR) in kidney and heart transplant biopsies correlated with increased DLEs representing activated mast cells, activated NK cells, and monocytes. Some findings were validated by single-cell/nucleus RNA-seq data from kidney and liver biopsies.

**CONCLUSION:**

The shared correlations of DLEs with rejection in all organs — e.g., CD4ma with TCMR and activated mast cells with ABMR — suggest novel aspects of rejection. These findings can guide the search for the in vivo leukocytes that correspond with the digital entities, with potential clinical applications.

**TRIAL REGISTRATION:**

ClinicalTrials.gov NCT01299168, NCT02670408, NCT02812290, NCT03193151.

**FUNDING:**

Genome Canada; Natera Inc.

## Abstract 2

We found a highly conserved digital profile of immune cells related to T cell mediated rejection and antibody-mediated rejection in kidney, heart, lung, and liver organ transplants.

## Introduction

Despite advances in organ transplantation, rejection-related graft loss remains problematic. In previous studies, we defined the molecular basis of the rejection states using genome-wide bulk transcriptomic analysis of organ allograft biopsies in the Molecular Microscope Diagnostic System (MMDx), which provided insights into the mechanisms of T cell–mediated rejection (TCMR) and antibody-mediated rejection (ABMR) (1–3). MMDx defined molecular TCMR in large cohorts of kidney, heart, lung, and liver transplant biopsies prospectively collected in registered clinical trials (4–7). All rejection manifested increased expression of pan-rejection transcripts, such as IFNG-inducible transcripts (e.g., CXCL11) and transcripts shared by T cells and natural killer (NK) cells (e.g., KLRD1) (8). TCMR in all organs displayed transcripts typical of activated effector T cells (e.g., IFNG) and classically activated macrophages (e.g., ADAMDEC1) (9). MMDx also defined a molecular ABMR state in kidney and heart transplant biopsies displaying increased expression of transcripts typical of NK cells (e.g., FGFBP2) and endothelial cells (e.g., ROBO4) (10–12), but found no similar molecular ABMR state in lung (13) or liver (14) transplants despite using similar methods. These bulk transcriptomic findings are now applied clinically to assess rejection in biopsies (2, 3, 15), and to support clinical trials of potential new therapies such as anti-CD38 for ABMR (16, 17).

Bulk transcriptomics does not identify the specific leukocyte populations underlying transplant rejection states — information that could be useful in the search for mechanisms that could be targeted by new therapies. Single-cell transcriptomics paired with highly specific cellular labeling and isolation technologies has the potential to provide insights into disease-specific leukocyte states and has mapped a clonally expanded CD8^+^ T cell population in TCMR (18) and clonally expanded B cells and plasma cells, compatible with a role for B cells as antigen-presenting cells in the TCMR inflammation process (19). Nevertheless, single-cell transcriptomics is limited in the number of biopsies that can be analyzed and is not applicable to large populations (20), making statistical analysis challenging, and may miss rare leukocyte classes of special interest, such as CD4^+^ memory T cells recently shown in mice to be critical for allorecognition (21).

To augment both single-cell and bulk transcriptomics studies, digital cytometry can be applied to estimate cellular composition or enrichment in tissues assessed by bulk transcriptomics using gene expression patterns derived from isolated cells (e.g., by flow cytometry or single cell transcriptomics workflows). A potential application of digital cytometry is to map digital leukocyte entities (DLEs) associated with rejection in all organ transplants, as well those selective for specific organs. This offers the possibility of examining shared cellular elements in large cohorts of allograft biopsies and provides additional endpoints for clinical trials and potential cellular targets for intervention.

The present study explored the DLEs associated with TCMR and ABMR in large cohorts of kidney (2), heart (3), lung (13), and liver (14) allograft biopsies preserved in RNA*later* (Figure 1). To discover the DLEs most specific for TCMR and ABMR, we excluded mixed and ambiguous cases. We assessed multiple cellular references (22–26) using CIBERSORTx (26–28) and compared their alignment with molecular TCMR and ABMR (26–28), and chose the LM22 reference because it was best aligned with rejection diagnoses and profiled the most extensive variety of DLEs (Supplemental Tables 1 and 2; supplemental material available online with this article; https://doi.org/10.1172/JCI208686DS1). Our rationale was that as a blood-based, tissue-agnostic, leukocyte reference, LM22 could profile the DLEs shared by rejection in all organ transplants, setting the stage for ultimately identifying the actual leukocytes and activation states in vivo that underlie these important disease processes. To partially validate the digital associations with TCMR and ABMR in the kidney, we collated publicly available kidney and liver allograft single-cell/nucleus RNA-seq (scsnRNA-seq) data with our own snRNA-seq cohort. Finally, we examined the evolution of the DLEs with standard-of-care (SOC) treatment and their association with risk of graft loss.

## Results

Kidney, heart, lung, and liver allograft biopsies were classified by their published automated rejection archetype diagnosis assigned by MMDx (4, 5, 13, 14) and locally assigned histology diagnoses. For ease of interpretation, and to focus our analyses on the most distinct and clinically relevant rejection states, molecular rejection classes were simplified to no rejection (NR), TCMR, and in kidney and heart transplants, ABMR. We also assessed histologically normal native kidney tissue from surgical nephrectomies for cancer (“native kidney”), acknowledging that these samples have aging and potentially other molecular abnormalities and should not be considered “normal” kidney tissue. We could not compare heart, lung, and liver transplants to native, untransplanted tissue from those organs due to the lack of suitably preserved tissue.

### Alignment of cellular references with rejection diagnoses.

Digital leukocyte composition was estimated by CIBERSORTx (26, 28) using the blood-based LM22 (26) and ImmunoStates references (13), as well as kidney-specific KTB18 (25), heart-specific Heart Cell Atlas (HCAv2) (23), lung-specific Human Lung Cell Atlas (HLCAv1.0) (22), and the liver-specific Liver Cell Atlas (LCA) references (Supplemental Table 1).The alignments of each reference with rejection diagnoses were compared using Silhouette scores (29), PERMANOVA r^2^, and reference principal component score associations with diagnoses (see Supplemental Methods for details). For each organ, the relevant metrics were then simplified as a composite score for ease of interpretation. LM22 had superior alignment with kidney, heart, and lung rejection diagnoses, and liver was not significantly different to ImmunoStates and the liver atlas (Supplemental Figure 1). The superior alignment of LM22 with MMDx rejection diagnoses is likely because transplant rejection is primarily associated with infiltration by leukocytes from blood in various activation states. However, all of these references offer unique perspectives and will be analyzed in detail in future studies, specifically regarding tissue-resident leukocytes and parenchymal cell injury.

### Assigning an estimated leukocyte burden to each biopsy.

To interpret digital leukocyte composition in light of the total leukocyte infiltrate among rejection groups, we used the expression of the pan-leukocyte gene *CD45* (*PTPRC*) to define an estimated leukocyte burden (ELB) (CD45_ELB_) in each biopsy (30). We later use CD45_ELB_ to estimate pseudoabundance scores to adjust CIBERSORTx estimates for the total leukocyte infiltrate in each biopsy.

Compared with native kidney tissue, kidney transplant biopsies with no molecular rejection or injury had a 2.7-fold increase in CD45_ELB_ (Table 1). When we included biopsies with molecular injury signals in the no-rejection group, the CD45_ELB_ was 4.19-fold higher than in native kidney tissue. The CD45_ELB_ estimates in heart, lung, and liver transplant biopsies classified as NR were similar to those in kidney biopsies with NR. The increased leukocyte burden in transplants diagnosed as NR compared with native kidney tissue presumably reflects injury-induced inflammation (31, 32), although some may reflect subthreshold alloimmune activity (31).

Biopsies with rejection had an increased leukocyte burden compared with biopsies with NR (Table 1). CD45_ELB_ was 5.8-fold higher in kidneys with TCMR (24-fold higher than native kidney); 6.8-fold higher in hearts with TCMR versus NR; 2.1-fold higher in lungs with TCMR, and 2.7-fold higher in livers with TCMR. CD45_ELB_ was higher in ABMR versus NR: 2.5-fold in kidney ABMR (10.1-fold higher than native kidney tissue) and 3.4-fold higher in heart ABMR.

All biopsies were performed as SOC, and the distribution of biopsies over time for each organ transplant reflected organ-specific biopsy practices (Figure 2A). The leukocyte burden was increased in all transplant biopsies but was independent of time of biopsy after transplant (Figure 2B). The increased leukocyte burden in TCMR and ABMR versus NR in kidney (Figure 2C) and heart (Figure 2D) transplants, and in TCMR versus NR in lung (Figure 2E) and liver (Figure 2F) transplants, was also relatively independent of time after transplant.

### The composition of digital leukocytes in transplant biopsies compared with native kidney tissue.

We designated the DLEs with a lower case “d” (e.g., “dCD4ma”) to distinguish them from actual leukocytes named in vitro. A complete list of cell types profiled by LM22 is provided in Supplemental Table 2. The 5 most abundant DLEs in the native kidney tissue were those designated as activated NK cells (dNKa), alternatively activated macrophages (dM2aa), CD8^+^ T cells (dCD8), regulatory T cells (dTreg), and naive B cells (dBn), together comprising 73% percent of all DLEs. Note the low percent dTreg in lungs. Despite the increased leukocyte burden in transplants versus native kidney tissue, these 5 DLEs continued to constitute much of the DLE population in nonrejecting transplant biopsies: kidney 63%, heart 68%, lung 47%, and liver 70% (Table 2). Thus, when the leukocyte burden increases, some elements of the DLE hierarchy are preserved.

### The composition of digital leukocytes in kidney transplant biopsies with TCMR or ABMR.

Compared with no rejection, both TCMR and ABMR displayed a percent increase in classically activated macrophages (dM1ca) and activated mast cells (dMCa), and a percent decrease in dTreg, activated dendritic cells (dDCa), and resting mast cells (dMCr) (Figure 3A).

The most striking feature of the digital leukocyte landscape of kidney TCMR was a percent increase in dCD4ma, dCD4Tfh, dTγδ, dCD8, and to a lesser extent dCDn. Memory B cells (dBm), plasma cells (dPC), dMCa, and eosinophils (dEosin) were also increased in TCMR. The percentages of NK cells (resting dNKr and activated dNKa), resting memory CD4 (dCD4mr), resting mast cells (dMCr), and dTreg were decreased in TCMR.

Kidney ABMR was associated with a percent increase in dNKa, monocytes (dMono), and resting DC (dDCr), as well as the panrejection dMCa and M1ca.

The percent of each DLE in kidney rejection was relatively unaffected by time after transplant (Figure 3B). However, in TCMR, the percent of dCD4ma was highest in biopsies 1- to 3-years after transplant, whereas dTγδ was highest in biopsies at later times after transplant. Later TCMR biopsies also had more dBm, dPC, and dMCr. The digital landscape of kidney ABMR showed little relationship to time of biopsy after transplant.

The DLEs associated with the Banff histology rejection diagnoses were like those in the molecular rejection groups (Supplemental Figure 2A), including changes with time after transplant. The dCD4ma were highest early after transplant, whereas dTγδ, dBm, and dPC were increased in late TCMR (Supplemental Figure 2B).

### Effect of SOC treatment on digital leukocyte composition.

Among the kidney transplants with TCMR in their first biopsy (biopsy 1), 84 had a follow-up biopsy (biopsy 2) after SOC treatment after a mean interval of 64 days. SOC treatment varied but always included high dose glucocorticosteroids. Compared with biopsy 1, the mean TCMR activity score was greatly reduced in biopsy 2, from 0.45 to 0.16 (*P* < 0.0001). SOC management resulted in a lower composition of all DLEs increased in TCMR (i.e., dCD4ma, dCD4Tfh, dTγδ, and dCD8, as well as dM1ca, dEosin), and an increase in DLEs decreased in TCMR (i.e., dMCr, dTreg, dNKa, and dM2aa), demonstrating that SOC management of TCMR was associated with at least partial suppression of TCMR molecular activity and reversal of the main DLE features of TCMR.

At present, only anti-CD38 monoclonals have been demonstrated to be effective in lowering histologic and molecular ABMR activity in randomized controlled trials (16, 33, 34). No anti-CD38 treatments were part of SOC treatment for ABMR in our cohort during the period in which these biopsies were collected, and the follow-up biopsies showed minimal decrease in digital leukocytes associated with all rejection and ABMR (data not shown).

### The composition of digital leukocytes in heart transplant biopsies with TCMR and ABMR.

The digital landscapes of heart transplant TCMR and ABMR were very similar to those in kidney transplants. All rejection showed an increase in dM1ca and dMCa. TCMR displayed an increased percent of the dCD4ma, dCD4Tfh, and dTγδ DLEs (Figure 4A). Heart ABMR was also like kidney ABMR, with an increased percentage of dMCa, dNKa, and dMono. As in kidney TCMR, the dCD4ma and dTγδ DLEs in heart TCMR changed with time post-transplant, with dCD4ma higher early post-transplant and dTγδ higher late post-transplant (Figure 4B).

The DLEs associated with ISHLT histologic TCMR and ABMR diagnoses were like those in the molecular rejection groups (Supplemental Figure 3A) with the TCMR relationships being most robust (i.e., dCD4ma, dCD4Tfh, dTγδ; Supplemental Figure 3B).

### Digital leukocyte composition in lung and liver transplant biopsies with TCMR.

In lung (Figure 5) and liver transplants (Figure 6), TCMR displayed increased panrejection changes (i.e., dM1ca) plus TCMR-selective changes (e.g., increased dCD4ma and dTγδ) similar to those in kidney and heart transplants with TCMR.

We also compared DLE relationships among histologic lung and liver diagnoses. For lungs, we compared LM22 estimates among ISHLT-A grade (i.e., acute cellular rejection grade). The DLE profile for ISHLT-A grades A3 and A4 (i.e., most intense acute cellular rejection) was like molecular TCMR (Supplemental Figure 4). The DLE profiles in liver had the weakest discrimination for TCMR across organs (discussed below), and this was also seen for histologic TCMR in liver. Nonetheless, the overall trends in hierarchy of DLE selective for molecular and histologic TCMR were similar, although often not significant when comparing histologic NR and TCMR (Supplemental Figure 5). For a more detailed description of the digital leukocyte landscape in lung and liver see the Supplemental Materials.

### scsnRNA-seq data support some digital leukocyte associations with rejection.

To support our digital findings, we annotated LM22 cell types to scsnRNA-seq data from 4 kidney cohorts and 1 liver cohort and compared resulting cell type compositions among rejection groups. We were unable to do this for heart and lung allografts, as there are no publicly available scsnRNA-seq data for these organs with diagnoses of rejection. For kidney, we integrated our own snRNA-seq cohort of 8 kidney allograft samples from 6 patients diagnosed with TCMR with publicly available scsnRNA-seq data from 3 independent cohorts of kidney transplant biopsies (BioStudies accession code E-MTAB-12051; GEO accession code: GSE195718; GEO accession code: GSE284742) (25, 35, 36) from patients diagnosed with NR, TCMR, and ABMR. After quality control, the combined kidney cohort included 15 NR, 6 TCMR, and 3 ABMR. For liver, we processed an independent cohort of liver allografts from patients diagnosed with NR and TCMR (GEO accession code: GSE256141). After quality control, the liver cohort included 3 NR and 14 TCMR. LM22 labels were assigned to leukocyte subsets using the SingleR algorithm (37).

The leukocyte profile for kidney TCMR and ABMR showed enrichment of M1ca in both TCMR and ABMR (i.e., all rejection), of CD4ma and CD4Tfh in TCMR, and of NKa in ABMR (Supplemental Figure 6, D and E). However, leukocytes corresponding with several DLEs could not be resolved convincingly by SingleR in the kidney cohort, including Tγδ, Tregs, dendritic cells, and mast cells. For liver, CD4ma were the only leukocytes associated with TCMR (Supplemental Figure 6I), with all others failing to meet statistical significance. The overall pattern in leukocytes correlated with liver TCMR versus NR were nonetheless supportive of the digital findings (Supplemental Figure 6J).

### A conserved digital leukocyte landscape of TCMR in all organs.

dCD4ma, dCD4Tfh, dTγδ, and dCD8 were associated with TCMR in all organs, as well as the pan-rejection dM1ca (Figure 7, A–D). Clustering of rank biserial correlations (rbcor) profiles showed strong selectivity of dCD4ma for TCMR in all organs (Figure 7E). The Spearman correlations of various DLE with TCMR archetype scores were also similar in all organs (Supplemental Table 3). Ranked based on the highest correlations with TCMR diagnosis across all organs, the top 6 DLEs positively correlated with TCMR were dCD4ma, dCD4Tfh, dTγδ, dCD8, dEosin (not in liver), and dPC, as well as the pan-rejection dM1ca. MCa and MCr showed variable TCMR associations.

The DLE with the largest CD45_ELB_-adjusted fold-changes (AFC) in TCMR was dCD4ma: kidney (55.4-fold), heart (53.8-fold), lung (28.3-fold), and liver transplants (30.5-fold) (Supplemental Table 4). The dTγδ also had large AFCs in TCMR versus NR in kidney (28.3-fold), heart (18.8-fold), and lung (25.8-fold), with a comparatively smaller AFC in liver transplants (5.1-fold). CD4Tfh was also increased in TCMR in kidney (9.9-fold), heart (8.8-fold), lung (4.4-fold), and liver (2.9-fold). dEosin, although rare, were increased in TCMR in kidneys, hearts, and lungs, consistent with earlier histology reports (38, 39).

To demonstrate the selectivity of the LM22 DLE profile for TCMR, we used percent DLEs to train a molecular classifier (trained on the molecular diagnoses) to predict the TCMR diagnoses. This predicted TCMR with high performance in all biopsy populations, with cross-validated area-under-the-receiver operator curves (AUCs) of 0.97 in kidney, 0.98 in heart, 0.96 in lung, and 0.81 in liver (Figure 7F).

Table 4 summarizes the percentage, adjusted fold-change, correlation with TCMR diagnoses, and correlation with TCMR archetype score for various T cell and DC DLEs. Although among the lowest percentages, dCD4ma had a high adjusted fold-change and correlations with TCMR, whereas dTreg correlated negatively with TCMR in kidney, heart, and liver transplants (although not in lung transplants, where the baseline percent dTreg was much lower). However, dTreg in TCMR had a weakly positive adjusted fold-change compared with NR, suggesting that the decreased percentage represents dilution (see discussion).

### The most selective digital leukocytes for ABMR in kidney and heart transplants.

dM1ca, dMCa, dNKa, and dMono all had high correlations with ABMR (Figure 8, A–C). These DLE also had high correlations with ABMR archetype scores (Supplemental Table 5). The strongest negative correlations were with dMCr and dTreg. The DLE most increased in ABMR by fold-change were dMCa (Supplemental Table 6).

Note that, although they did not correlate with ABMR, TCMR-related T cells (dCD4ma, dT**γ**δ, dCD4Tfh, dCD8) were increased by adjusted fold-change in ABMR about as much as the expansion of the leukocyte burden.

As with TCMR, the DLE profiles strongly predicted ABMR in the biopsy populations, with AUCs of 0.93 in kidney and 0.92 in heart (Figure 8D).

Table 5 compares the percentage, fold-changes and correlations with kidney and heart ABMR for digital leukocytes representing mast cells, dNKa, and dendritic cells. The fold-change and correlations of dMCa, dNKa, and dDCr were increased in ABMR, whereas those for dMCr, dNKr, and dDCa were decreased.

### Digital leukocytes common to all rejection.

We characterized the correlations of DLE in NR versus all rejection (including possible rejection) in kidney and heart transplants as an estimate of the pan-rejection changes (Figure 9, A–C). The DLEs most correlated with all rejection were dM1ca and dMCa. Conversely, the DLEs anticorrelated with rejection were dMCr and dTreg. As with TCMR and ABMR, the DLE profiles strongly predicted all rejection in the biopsy populations, with AUCs of 0.95 in kidney and 0.98 in heart (Figure 9D).

### Kidney allograft survival is associated with the molecular rejection states predicted by digital leukocyte composition.

We compared Kaplan-Meier estimates of 3-year postbiopsy kidney graft loss stratified by MMDx rejection groups to survival in rejection groups predicted using LM22 composition (Figure 10, A and B). Median follow-up time within the survival cohort was 539 days. Rejection diagnosed by the LM22-based classifier provided slightly stronger associations with graft loss compared with the MMDx diagnoses, indicating that reclassification assisted by digital cytometry estimates improved estimates of survival in our cohort.

We then studied how well DLE measurements alone (i.e., independent of rejection diagnoses) were able to predict graft loss. We trained a 10-fold cross-validated random survival forest using LM22 composition as features, which produced poor performance (C-index_kidney_ = 0.66; C-index_heart_ = 0.68; C-index_lung_ = 0.67), indicating that DLE composition alone provides limited value for predicting graft loss postbiopsy. Thus, the DLEs predicted the disease states associated with survival, but survival estimates based on DLE composition alone did not strongly predict survival, which is expected given that postbiopsy survival is dictated primarily by parenchymal injury, even within the rejection states (40, 41).

Because of the strong association between the rare dCD4ma and dTγδ with TCMR, we specifically explored the associations of these individual DLEs by stratifying them as present or absent, and found the presence of dCD4ma and dTγδ was associated with poorer outcome (Figure 10, C and D).

## Discussion

This project aimed to define the DLEs shared by organ transplants with TCMR and ABMR with the ultimate goal of understanding the in vivo leukocyte populations and activation states most associated with clinical rejection stages. We applied digital cytometry to bulk microarray transcriptomics data from 8,422 organ transplant biopsies (kidney, heart, lung, and liver), excluding mixed and ambiguous cases for this first analysis, comparing the DLEs to molecular and histologic rejection diagnoses. Because of the pan-organ nature of our mission, we focused on the blood-based LM22 leukocyte reference, which allowed annotation of many DLEs and activation states of special interest in rejection. LM22 has been validated in various tissues (42, 43), and we found that LM22 had better alignment with the rejection states than other references. All rejection had increased percentages of dM1ca and dMCa and (except in lung) decreased percentages of dTregs. TCMR in all organs was highly associated with increased percentages of the rare dCD4ma and dTγδ, followed by dCD4Tfh, all more strongly TCMR associated than the more abundant dCD8. These findings were consistent across molecular and histologic rejection diagnoses in all organs. The selectivity of dCD4ma for TCMR in kidney and liver was supported by scsnRNA-seq findings. However, Tγδ could not be resolved in the scsnRNA-seq kidney cohort, and while measured higher by composition in liver TCMR scRNA-seq, was not statistical significant, and will require further investigation. The conserved digital landscape of ABMR in kidney and heart transplants featured the expected selective increases in dNKa, dMono, and the pan-rejection dM1ca and dMCa. ScsnRNA-seq found increased NKa in ABMR but was unable to annotate mast cells. We propose that these DLE landscapes represent insights into the conserved leukocyte relationships in all of the major rejection states across all organ transplants. However, their true relationship to actual in vivo leukocytes and activation states must be definitively established in future studies, and we suspect that in vivo states may not fully align with the in vitro assigned entities. Moreover, future single organ studies will require investigation of organ-specific parenchymal cells and resident leukocytes not represented in blood.

Changes in leukocyte burdens and in relative fold-changes in DLEs could reflect a variety of factors, including infiltration by circulating leukocytes, transitions between resting and activation states, mitosis, and cell death. We know that in transplants with TCMR, some T cells (18), B cells, and PC (44) clonally expand, probably primarily in secondary lymphoid organs but potentially also in the transplant. Transitions may affect both resident leukocytes (e.g., MCr to MCa) and infiltrating leukocytes (M2aa to M1ca; Bn to Bm to PC). Cognate CD4 memory cells are probably activated by engaging antigen-presenting cells in the rejecting organs (e.g., CD4n or CD4mr to CD4ma), having homed to the graft after initial activation/clonal expansion in secondary lymphoid organs.

The dCD4ma DLE increased 28-to-55-fold (adjusted) across organs and had the strongest correlation with the TCMR in all organ transplants, despite being only 2%–3% of DLE in TCMR. This echoes the recent experimental results in mice, indicating the key role of CD4 effector memory T cells in allorecognition, licensing antigen-presenting cells (APCs) to recruit and activate other cognate T cells and B cells and triggering innate immunity programs (21). DLE correlations with TCMR suggest that a small population of cognate memory CD4^+^ T cells previously clonally expanded by donor alloantigen on host and/or donor APCs in secondary lymphoid organs (some perhaps arising from heterologous immunity) (45, 46) play a critical role in orchestrating the TCMR infiltrate. We propose that the cognate activated CD4ma or their precursor CD4mr home to the graft, cross the microcirculation endothelium (47) to enter the interstitium, engage and are activated by APC, and license the APC (21) to recruit more cognate CD4 and CD8 cells, activating M1ca and MCa and recruiting the eosinophils often observed in TCMR infiltrate (38, 48–51). dPC, and less consistently dBm, were increased in TCMR in all organs, consistent with the evidence that Bm can serve as APCs to sustain chronic T cell–mediated inflammation (19, 52) and interact with CD4Tfh to mature to PC, contributing to the emergence of DSA and ABMR. This possibility is supported by the recent finding of clonally expanded PC and Bm in TCMR (19, 52).

The 5-to-28-fold increases in dTγδ in TCMR in all organ transplants and its strong correlations with TCMR, particularly at later times after transplant, offers a new dimension to explore in the pathogenesis of TCMR, with the caveat that scsnRNA-seq failed to identify Tγδ in our kidney cohort. Tγδ are a diverse population of T cells with properties that overlap those of NK cells but are not usually considered to be involved in alloimmune responses because γδ receptors are not MHC restricted or known to recognize MHC alloantigens (53). However, there is increasing interest in the potential roles of Tγδ in alloimmunity (54). The association of dCD4ma with early TCMR and of dTγδ with late TCMR in kidney and heart biopsies suggests possible evolution of the host cognate T cell response in the alloimmune reactions. In organ transplant, time has a powerful effect on TCMR, which is frequent in the early years after transplant but rare at later times as ABMR becomes predominant (55).

The shared digital leukocyte landscape of kidney and heart transplants with ABMR supports the key role for activated NK cells in ABMR, consistent with recent studies showing that suppression of blood NK cells by anti-CD38 monoclonal antibody is associated with reduction in ABMR activity in kidney transplants despite persistence of donor-specific antibody (16, 34). In the present study, dNKa correlated with ABMR, but the increase in the adjusted fold-change in dNKa was only modest. This was as expected, because the NKa cells associated with ABMR are only a minority of the NK population, namely the circulating CD56dim CD16a-positive NK cells in the capillaries activated by their CD16a Fc receptors engaging endothelium-bound alloantibody (16, 56, 57), and perhaps also sensing “missing self” (16). The surprising percentages of dNKa in the nontransplant nephrectomy kidney tissue (albeit with a very low total leukocyte burden) and in nonrejecting kidney and heart transplant biopsies must be interpreted against the heterogeneity of NK cells. NK, Tγδ, and NKT leukocytes are known to share many features. For example, both dNKr and dNKa, as profiled by LM22, express T cell δ receptor gene mRNA (TRDC), which is highly correlated with ABMR activity in kidney transplants (10) and was expressed in the NK cells used in the LM22 study (26). NK cells are very diverse (58), including both tissue resident and circulating NK cells, and many factors regulate their activation positively and negatively (59–62).

The unexpectedly strong association of dMCa with rejection, particularly ABMR, along with a decrease in dMCr, suggests that ABMR activates resting resident mast cells, perhaps via the cytokine milieu that we believe also activates the M1ca. Mast cell activation could be a source of stress on the endothelium and other parenchymal elements in ABMR, potentially contributing to progression to organ failure and amenable to targeted intervention. Additionally, the association of dMono with ABMR in kidney and heart biopsies, and of eosinophils with TCMR in all organs, further supports the role of the innate immune response in rejection and may offer new targets interventions.

The molecular mechanisms that injure parenchyma in rejection are unknown but are currently being explored in scsnRNA-seq studies and spatial transcriptomics of parenchymal, matrix, and endothelial cells (35). Efforts to disrupt graft injury in TCMR in mice by knocking out individual genes such as granzymes, perforin, Fas-Fas ligand, IFNG, and other cytokines have not been successful (63). We have been mapping the epithelial injury response in kidney TCMR, which in part resembles other forms of acute kidney injury (40), though likely with unique elements as well (31). It is crucial to define the details and consequences of the cognate engagement of individual lymphocytes such as CD4ma or NKa with graft cells.

Despite the strong conservation of the digital leukocyte landscape of TCMR in all organ transplants and of ABMR in kidney transplants, there were differences among rejecting organ transplants, as expected given differences in parenchymal mass and innate immune features. The lack of a molecular ABMR in liver and lung transplants could reflect the greater mass of liver and lung, making them more resistant to the limited effector mechanisms such as blood NK cells (56, 64). The smaller increase in leukocyte burden and LM22 DLEs in TCMR in liver versus kidney and heart could also reflect the greater mass (> 10-fold larger than kidneys or hearts) and unique antigen-presenting structures of liver (65), echoing the general belief that TCMR is less aggressive in liver transplants (66). The digital landscape of lung transplants differed from the other organs in having the lowest percentage of dTreg.

Given the recent recognition of Tregs by the Nobel prize in medicine (https://www.nobelprize.org/prizes/medicine/2025/press-release/), dTreg are of interest because of their lower percent representation in rejection. This decline is consistent with dilution rather than decreased absolute number, since the estimated fold-change in dTregs was weakly positive (2-to-4-fold in TCMR and 1.5-fold in ABMR) when adjusted for the leukocyte burden. In unpublished studies (67), we have found that injury reduces the percentage of dTreg in kidney transplant biopsies. However, leukocytes corresponding to dTregs could not be resolved in the kidney and liver scsnRNA-seq cohorts, possibly reflecting the diversity of Tregs (18).

Despite the encouraging scsnRNA-seq findings, the principal limitation of this study is the need for comprehensive orthogonal validation of relationships of DLE to actual in vivo leukocyte and activation states by alternative methods, particularly for low-abundance leukocytes like CD4ma. Previous single-cell studies of human transplant biopsies (18, 25, 35, 56, 68) have not annotated CD4ma and Tγδ, probably reflecting the rarity of these leukocytes and of TCMR biopsies suitable for single-cell studies. LM22 also lacks definition of subsets and activation states of CD8^+^ T cells, Th1, Th2, and Th17, and monocyte subsets. Indeed all cellular references are currently challenged to capture to the full repertoire of leukocytes and activation states.

The present findings illustrate the power of combining bulk transcriptomics with digital cytometry for understanding clinically important disease states. The digital landscape has many potential implications for basic experimental studies and for understanding responses to therapy, but the relationship of each DLE to leukocytes, as defined in vitro, must be established. In terms of next steps, deeper exploration within each organ is necessary, including profiling tissue- and transplant-specific cellular references (e.g., KTB18 for kidney), as well as exploration of clinically ambiguous states such as borderline TCMR and the molecular NK-cell enriched rejection state (NKRL) in lung and liver transplants. Digital cytometry offers opportunities in clinical diagnostics and clinical trials (e.g., assessing heterogeneity in TCMR and ABMR, monitoring the effects of treatment, and detecting residual abnormalities after treatment). The DLEs most selective for TCMR and ABMR suggest potential new cellular targets for therapy (e.g., CD4ma for TCMR), and the relative expression of individual DLEs in a specific case (e.g., CD4ma vs Tγδ versus CD4Tfh in TCMR and NKa versus monocytes versus mast cells in ABMR) guide selection of targeted therapies. Admittedly, targeting activated subpopulations such as CD4ma with monoclonals, fusion proteins, or CART therapy will be challenging because activation states can be transient, and not necessarily represented by membrane markers (69–71). Nevertheless, specifically targeting small leukocyte populations while sparing nontarget populations currently suppressed by nonselective therapies such as high-dose corticosteroids and generalized T cell depleting agents (72) is inherently attractive for reducing infection and malignancy risks. Indeed, the concept of seeking small cell populations with strong associations with rejection states recalls how new therapies for ABMR emerged, where NK cells were first found to be associated with ABMR (56) and are now being targeted by therapies (16, 34).

## Methods

### Sex as a biological variable.

The male and female status of the donors and recipients were recorded in the original status and have no known impact on the presented findings.

### Study populations.

The MMDx studies (INTERCOM/INTERCOMEX: ClinicalTrials.gov #NCT01299168, Trifecta-Kidney: ClinicalTrials.gov #NCT04239703; INTERHEART: ClinicalTrials.gov #NCT02670408; INTERLUNG: ClinicalTrials.gov #NCT02812290; INTERLIVER: ClinicalTrials.gov # NCT03193151) are prospective multicenter studies of consenting patients involving 74 investigators from 67 transplant institutions (Supplemental Table 7) under local IRB-approved protocols as previously described (2, 3, 13, 14). The biopsy rejection phenotype classifications along with patient demographics have been published (2, 3, 13, 14).

Additionally, to assess if SOC resulted in a reduction in the composition of digital T cell leukocytes, a sub-cohort of 82 patients from INTERCOM/INTERCOMEX initially diagnosed with TCMR in their first biopsy and who had a follow-up biopsy were isolated.

### Molecular diagnostics.

Biopsies were diagnosed molecularly by MMDx with analyses focussing on NR, TCMR, and ABMR archetypal assignments. For Kidney, NR included NR, TCMR included TCMR1 and TCMR2, and ABMR included EABMR, FABMR, and LABMR archetype assignments. For heart, NR included NR, TCMR included TCMR1 and TCMR2, and ABMR included EABMR and FABMR. For lung, NR included NR and NRloSFT, and TCMR included TCMR (13). For liver, NR included NR, and TCMR included TCMR (14). Note that there is no MMDx phenotype for ABMR in lung or liver. All other biopsies were excluded, resulting in a total of 4961 kidney transplant biopsies (n_NR_ = 2842; n_TCMR_ = 627; n_ABMR_ = 1492), 3079 heart transplant biopsies (n_NR_ = 2568; n_TCMR_ = 153; n_ABMR_ = 358), 652 lung transplant biopsies (n_NR_ = 556; n_TCMR_ = 96), 540 liver transplant biopsies (n_NR_ = 415; n_TCMR_ = 125).

### Histologic diagnostics.

Biopsies were additionally categorized by local SOC histologic assessment, resulting in a total of 3165 kidney transplant biopsies (n_NR_ = 2547; n_TCMR_ = 172; n_ABMR_ = 446), 833 heart transplant biopsies (n_NR_ = 638; n_TCMR_ = 119; n_ABMR_ = 76), 517 lung transplant biopsies (n_A0_ = 314; n_A1_ = 126; n_A2_ = 69; n_A3_ = 5; n_A4_ = 3), 263 liver transplant biopsies (n_NR_ = 205; n_TCMR_ = 58).

### Digital cytometry.

Cellular compositions were estimated with CIBERSORTx (26–28). We focused on the LM22 signature matrix, which is comprised of 547 marker genes depicting 22 human immune leukocyte types (Supplemental Table 2). We also explored an additional blood matrix, ImmunoStates, as well as organ-specific matrices for kidney (KTB18), heart (Heart Cell Atlas v2; HCAv2), lung (Human Lung Cell Atlas v1.0; HCLAv1.0), and liver (Liver Cell Atlas; LCA). For LM22 and ImmunoStates (i.e., microarray-based matrices), bulk transcriptomics data were adjusted using B-mode batch correction to account for cross-platform technical variability. For KTB18, HCAv2, HCLAv1.0, and LCA (i.e., RNA-seq-based matrices), signature matrices were adjusted using S-mode batch correction. *P* values for CIBERSORTx estimates were obtained using 1000 permutations, and samples having *P* < 0.05 were excluded from analyses involving leukocyte compositions. For LM22, this resulted in a total of 4898 kidney transplant biopsies (n_NR_ = 2782; n_TCMR_ = 625; n_ABMR_ = 1491), 3073 heart transplant biopsies (n_NR_ = 2566; n_TCMR_ = 151; n_ABMR_ = 356), 652 lung transplant biopsies (n_NR_ = 556; n_TCMR_ = 96), 538 liver transplant biopsies (n_NR_ = 414; n_TCMR_ = 124).

### Estimated leukocyte burden and pseudoabundance scores.

Relative leukocyte burdens (CD45_ELB_) for each biopsy were estimated via expression of *CD45*/*PTPRC* (30). For each organ, a reference CD45_ELB_ was defined as the mean CD45_ELB_ in NR biopsies. A reference CD45_ELB_ was also defined for normal (non-transplanted) kidney tissue. For each biopsy, fold-changes in the respective CD45_ELB_ reference for its organ type were calculated. DLE pseudo-abundance scores were calculated by multiplying unadjusted percentages by the CD45_ELB_ fold-change. This pseudo-abundance score assumes each biopsy and leukocyte type shares the same denominator for total leukocyte counts.

### Single-cell/nucleus RNA sequencing analyses of kidney and liver allograft biopsies.

A meta-analysis of de-novo single nucleus kidney allograft data combined with three public kidney cohorts, and one public liver allograft cohort, was carried out to compare re-annotated LM22 leukocytes across NR, TCMR, and ABMR diagnoses. Complete details are provided in the Supplemental methods.

### Analysis of leukocyte composition.

Leukocyte compositions were presented as percent composition; fold-changes in percent in TCMR or ABMR versus NR; pseudo-abundance fold-change for digital leukocyte estimates (necessary because a percent decline may reflect dilution by infiltrating cells rather than a reduction in absolute cell numbers); rank-biserial correlations (rbcor) of the percent composition in TCMR vs ABMR + NR, ABMR vs TCMR + NR, and NR vs TCMR + ABMR; and Spearman correlation coefficients (SCC) of each DLE percent with the molecular rejection archetype scores in the entire biopsy population.

### Statistics.

For bulk transcriptomics and scsnRNA-seq data, differences in DLE type fraction among rejection groups were analyzed using Bayes moderated *t* tests with arcsin-transformed proportions (i.e., the Propeller test) (73). rbcor were calculated using DLE percentages and rejection groupings. Time series analyses were done via the binscatter method (74, 75). Fold-changes in DLE percentages in rejection groups compared with NR are represented as the mean (95% confidence interval) from 1,000 bootstrap resamples. A multiclass generalized linear model with least absolute shrinkage and selection operator (LASSO) for feature reduction (glmnet) was trained to assess the predictive performance of estimated DLE for rejection states. All classification was carried out with the tidymodels framework in R (76). Kaplan-Meier estimates were calculated using the ‘survival’ package in R. For measuring the effect of SOC on patients with kidney allografts diagnosed with TCMR, mean differences in digital leukocyte compositions were compared between index (B1) and follow-up (B2) using permutation tests.

All statistical analyses were carried out in R version 4.3.3. All tests were 2-tailed and a *P* value less than 0.05 was considered significant.

### Study approval.

5,086 kidney transplant biopsies were collected from 3,995 patients through the MMDx-Kidney studies, as well as through the MMDx service laboratory in Portland, Oregon, USA (Kashi Laboratories, submitted as anonymized files for this study) (4). 3,230 heart transplant endomyocardial biopsies were collected for clinical indications or for protocol/surveillance from consenting patients as per local SOC (5). 896 lung transplant transbronchial biopsies (TBBs) from 672 patients were taken in patients undergoing TBB for clinical indications or surveillance (13), as per SOC. 765 liver transplant biopsies were collected from 680 consented patients (per local standards at each center) (14). These studies adhere to the Declaration of Helsinki. All biopsies were collected with informed consent per IRB review at each local center and approved in Edmonton by the University of Alberta (Pro00022226, Pro00022227, Pro00048176, Pro00022225). The clinical and research activities being reported are consistent with the Principles of the Declaration of Istanbul as outlined in the ‘Declaration of Istanbul on Organ Trafficking and Transplant Tourism’.

Four normal kidney samples taken from nephrectomies for cancer, collected from 4 patients, were used as a control set to assess digital leukocyte composition (77).

### Data availability.

For bulk transcriptomics data, CEL files are available at Gene Expression Omnibus (Kidney [*N* = 5086]: GSE275126; Heart [*N* = 3230]: GSE262742; Liver [*N* = 765]: GSE277334; and Lung [*N* = 895]: GSE303535). For de-novo single nucleus RNA-seq, the raw counts and metadata from 8 TCMR samples are available at Gene Expression Omnibus GSE346749. Values for all data points in graphs are reported in the Supporting Data Values file.

## Manuscript information

### Author contributions.

PTG was responsible for data analysis and interpretation, writing, editing, and reviewing the manuscript. LMSG planned, conducted and supervised the snRNA-seq experiments and reviewed the manuscript. LJ was responsible for data analysis and interpretation. CH planned, conducted and supervised the snRNA-seq experiments, and edited and reviewed the manuscript, and was responsible for data interpretation. PFH was the principal investigator, edited, and reviewed the manuscript, and was responsible for data interpretation and study design. The INTERCOMEX, INTERHEART, INTERLUNG, and INTERLIVER Investigators were responsible for providing biopsies from consenting patients.

### AI disclosure.

The authors declare that no AI tools were used in the original research for or writing of this article.

### Conflict of interest.

PFH is a professor of medicine at the University of Alberta and Director of the Alberta Transplant Applied Genomics Centre. In addition, he is Chief Medical Officer at Transcriptome Sciences Inc. (TSI), a spin-off research company of the University of Alberta dedicated to developing molecular diagnostics. He is a consultant to Kashi Clinical Laboratories, Natera Inc., Biogen, and Argenx BV.

### Funding.

This study was supported by the following:

- One Lambda Inc./Thermo Fisher Scientific (to the Microarray biopsy assessment project).
- Research grant #49 from Mendez National Institute of Transplantation Foundation (MNITF) (to the Microarray biopsy assessment project).
- Natera Inc. (to the Microarray biopsy assessment project).
- Deutsche Forschungsgemeinschaft DFG (grant HI 2238/2–1) (to CH).
- Ministry for Science and Culture of Lower Saxony as project of the Center for Organ Regeneration and Replacement (CORE).
- Transplant Center, Hannover Medical School.
- A clinician scientist grant from the German Society of Internal Medicine (Deutsche Gesellschaft für Innere Medizin – DGIM) (to LMSG).

## Supplementary Material

Supplemental data

ICMJE disclosure forms

Supporting data values

## Figures and Tables

**Figure 1:**
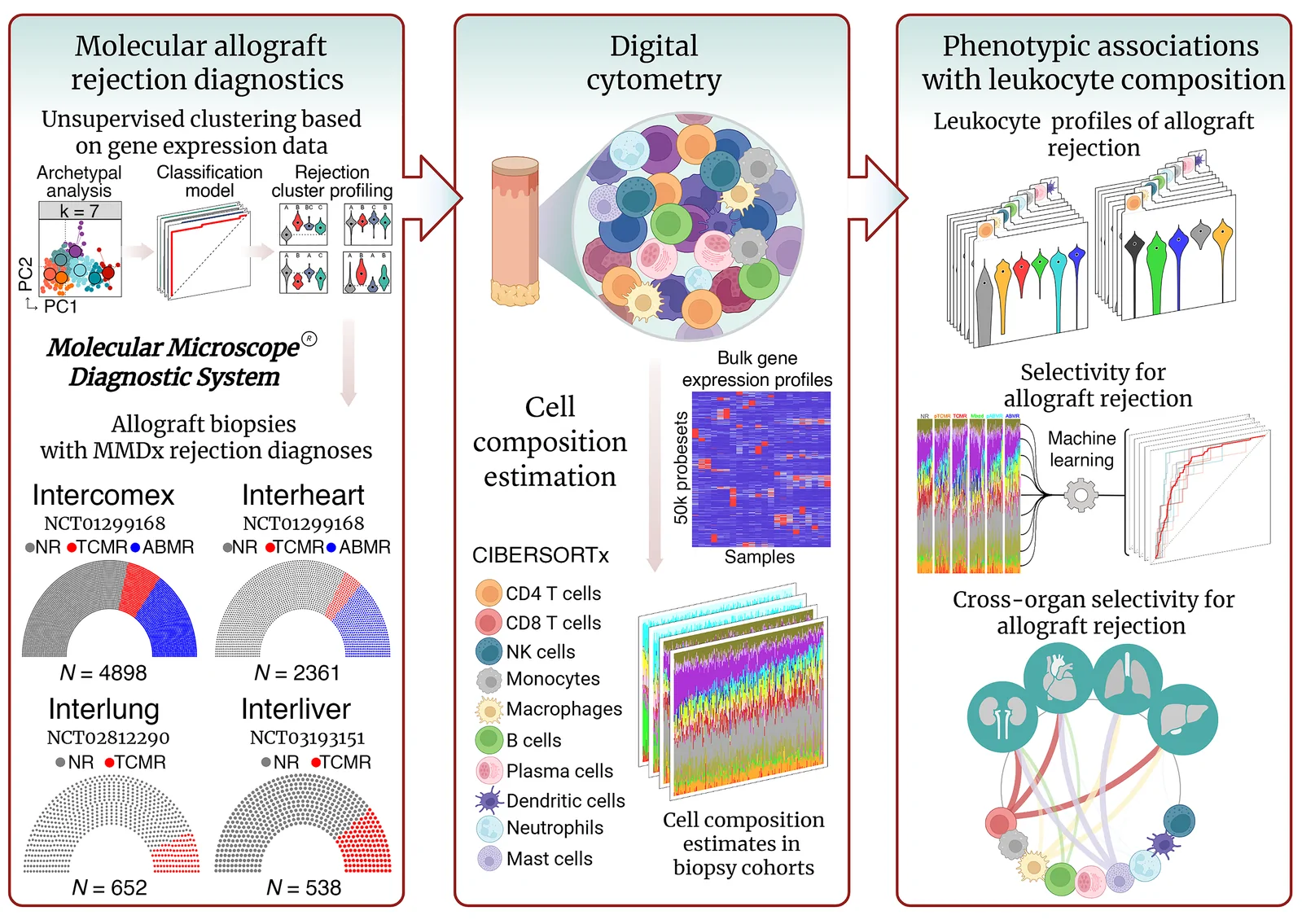
Study overview.

**Figure 2:**
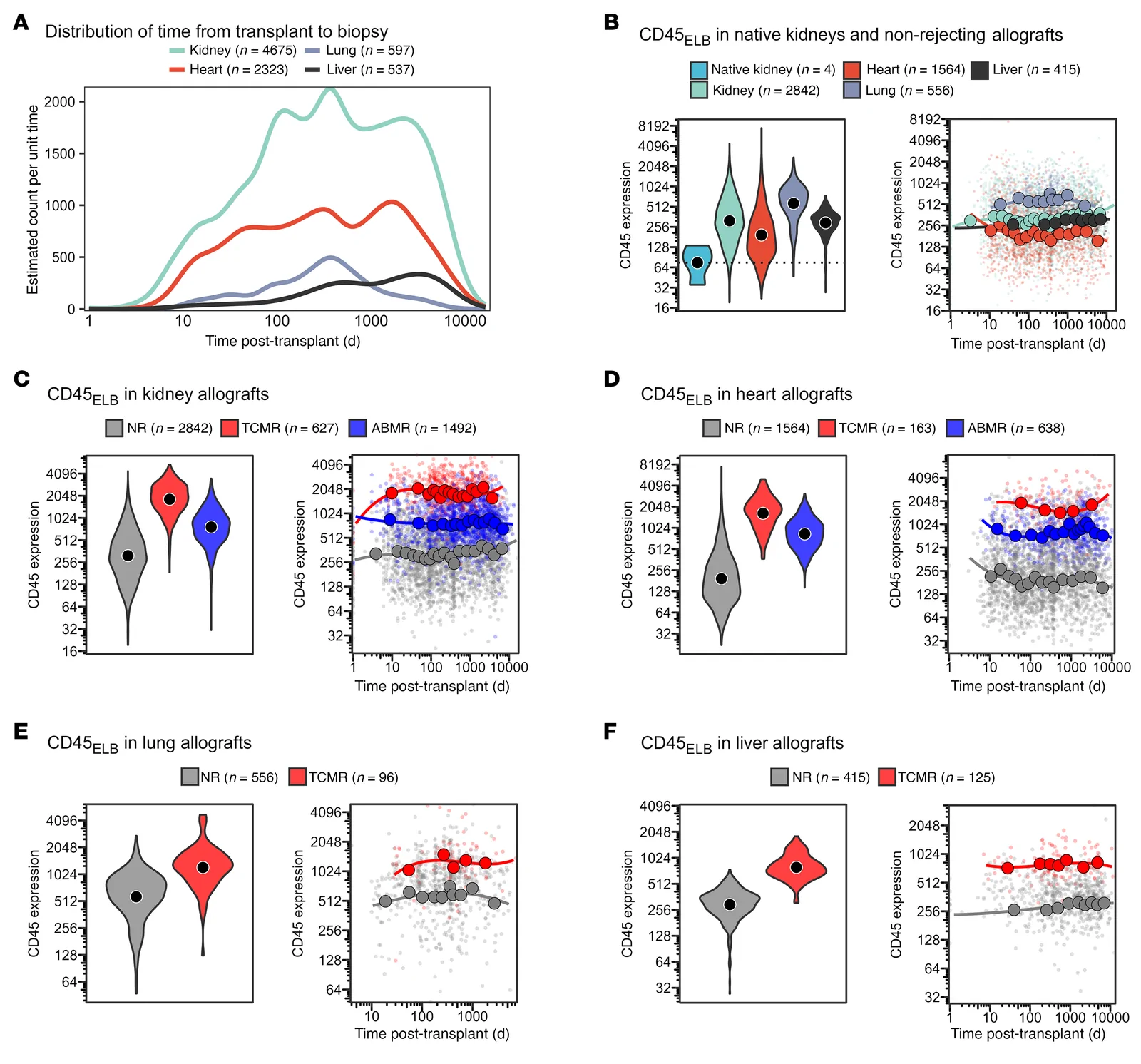
Distribution of biopsies and estimated leukocyte burden. (**A**) Curves depict kernel density estimates of the distribution of biopsies after transplant for kidney, heart, lung, and liver allograft populations. (**B**) estimated leukocyte burdens (CD45_ELB_) and time courses in normal kidney tissue and nonrejecting kidney, heart, lung, and liver allograft tissue. Expression values from microarray analyses (Affymetrix) are robust multiarray average (RMA)-normalized luminosity and are arbitrary. Colored blobs in violin plots depict the distribution of CD45_ELB_ for each group. Solid black circles in violin plots denote the mean CD45_ELB_ for each group. Small transparent circles in time series plots depict CD45_ELB_ in individual biopsies. Large colored circles depict binned CD45 expression using the binscatter method. Curves depict a polynomial fit of CD45_ELB_. (**C**–**F**) estimated leukocyte burdens and time courses in kidney, heart, lung, and liver allograft tissue by molecular diagnoses of T cell–mediated and antibody-mediated rejection. Colored blobs in violin plots depict the distribution of CD45_ELB_ for each group. Solid black circles in violin plots denote the mean CD45_ELB_ for each group. Small transparent circles in time series plots depict CD45_ELB_ in individual biopsies. Large colored circles depict binned CD45_ELB_ using the binscatter method. Curves depict a polynomial fit of CD45_ELB_.

**Figure 3:**
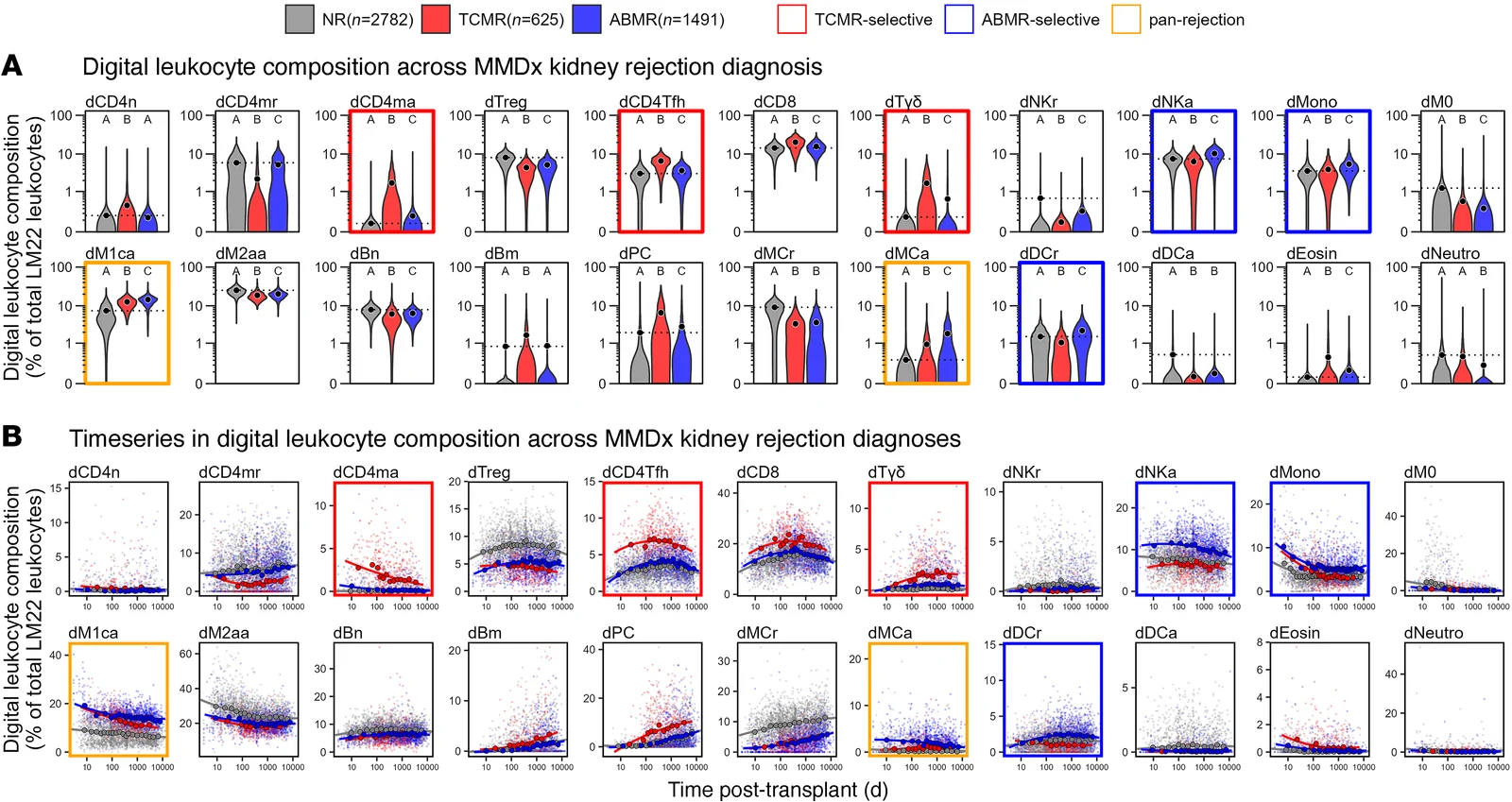
Composition and longitudinal changes in digital leukocytes in kidney transplant biopsies. Digital leukocyte composition was estimated using the CIBERSORTx algorithm and LM22 signature matrix. (**A**) Group-wise comparisons are illustrated as violin plots. Black circles in violin plots depict the mean percent composition by group. Compositional differences in digital leukocytes among rejection groups was analyzed using Bayes moderated *t* tests with arcsin-transformed proportions. Letters at the top of the plot depict a compact letter display of all-pairwise post hoc testing, where groups that share the same letter(s) are not different from one another. (**B**) Time series of estimate leukocytes posttransplant. Small transparent circles depict estimated leukocyte percentages in individual biopsies. Large colored circles depict binned estimated leukocyte percentages using the binscatter method. Curves depict a polynomial fit of estimated leukocyte percentages.

**Figure 4:**
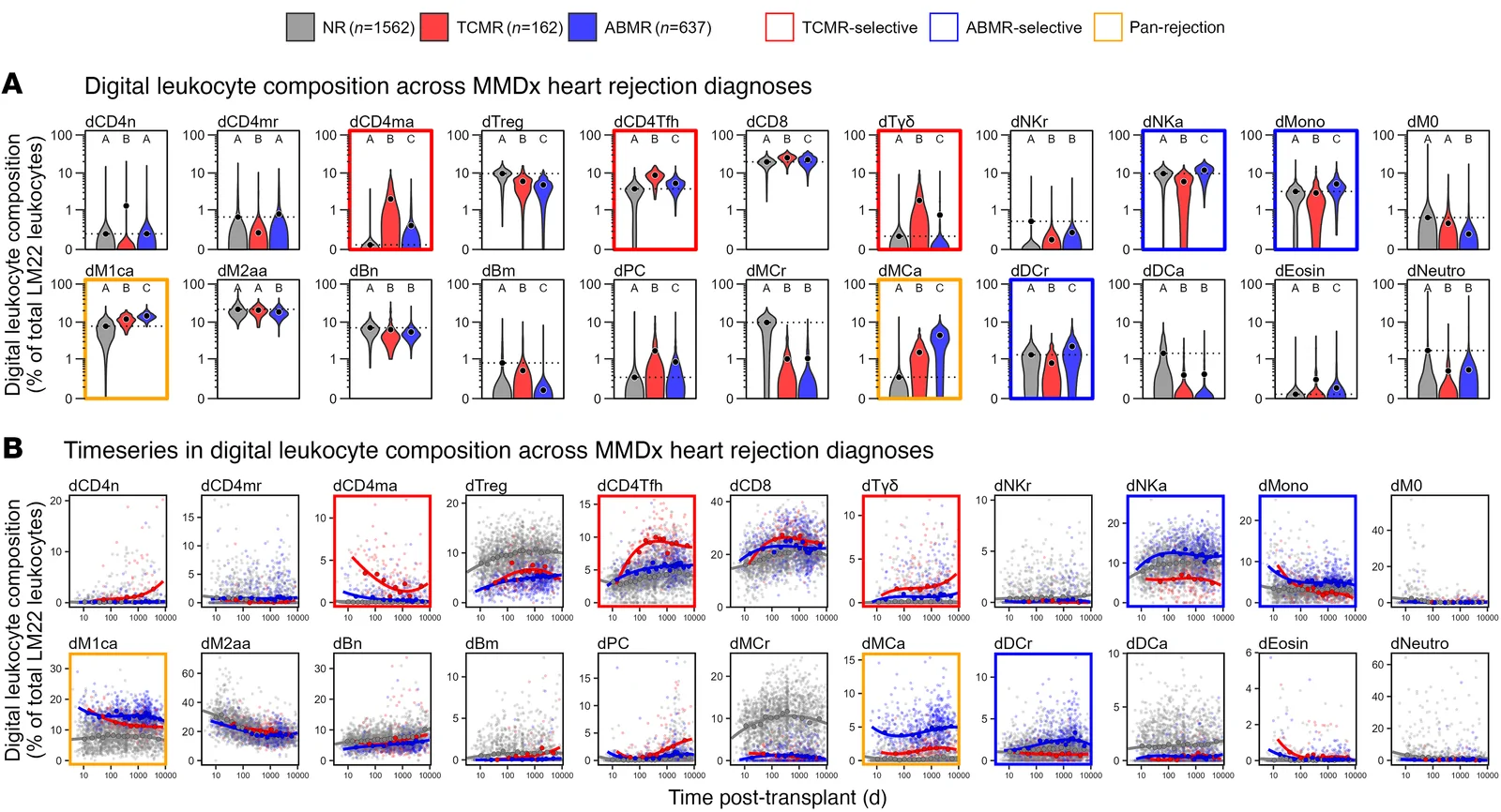
Composition and longitudinal changes in digital leukocytes heart transplant biopsies. Leukocyte profiles were estimated using the CIBERSORTx algorithm and LM22 signature matrix. (**A**) Group-wise comparisons are illustrated as violin plots. Black circles in violin plots depict the mean percentage composition by group. Compositional differences in digital leukocytes among rejection groups was analyzed using Bayes moderated *t* tests with arcsin-transformed proportions. Letters at the top of the plot depict a compact letter display of all-pairwise post hoc testing, where groups that share the same letter(s) are not different from one another. (**B**) Time series of estimate leukocytes after transplant. Small transparent circles depict estimated leukocyte percentages in individual biopsies. Large colored circles depict binned estimated leukocyte percentages using the binscatter method. Curves depict a polynomial fit of estimated leukocyte percentages.

**Figure 5:**
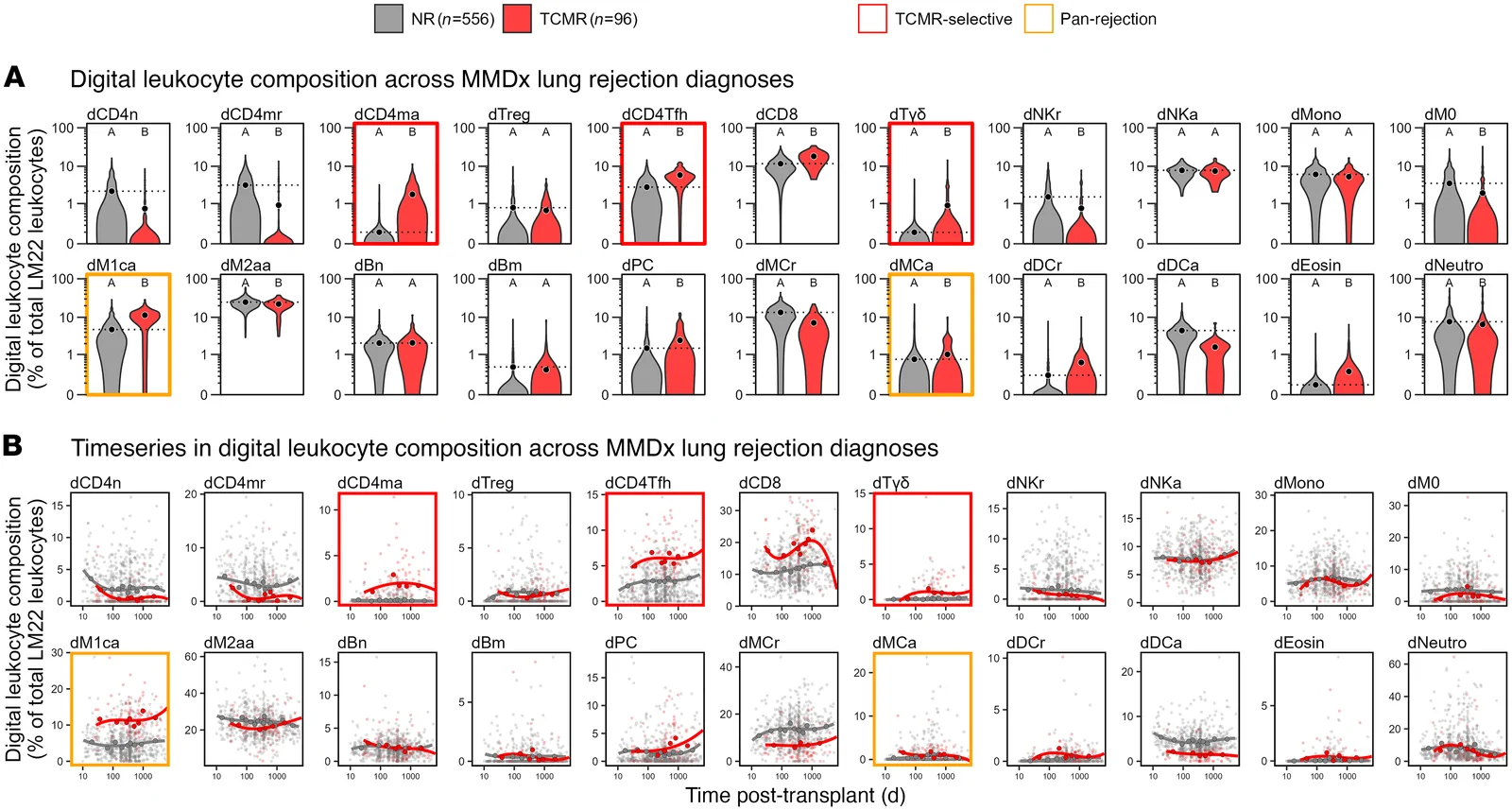
Composition and longitudinal changes in digital leukocyte lung transplant transbronchial biopsies. Leukocyte profiles were estimated using the CIBERSORTx algorithm and LM22 signature matrix. (**A**) Group-wise comparisons are illustrated as violin plots. Black circles in violin plots depict the mean percent composition by group. Compositional differences in digital leukocytes among rejection groups was analyzed using Bayes moderated *t* tests with arcsin-transformed proportions. Letters at the top of the plot depict a compact letter display of all-pairwise post-hoc testing, where groups that share the same letter(s) are not different from one another. (**B**) Time series of estimate leukocytes after transplant. Small transparent circles depict estimated leukocyte percentages in individual biopsies. Large colored circles depict binned estimated leukocyte percentages using the binscatter method. Curves depict a polynomial fit of estimated leukocyte percentages.

**Figure 6:**
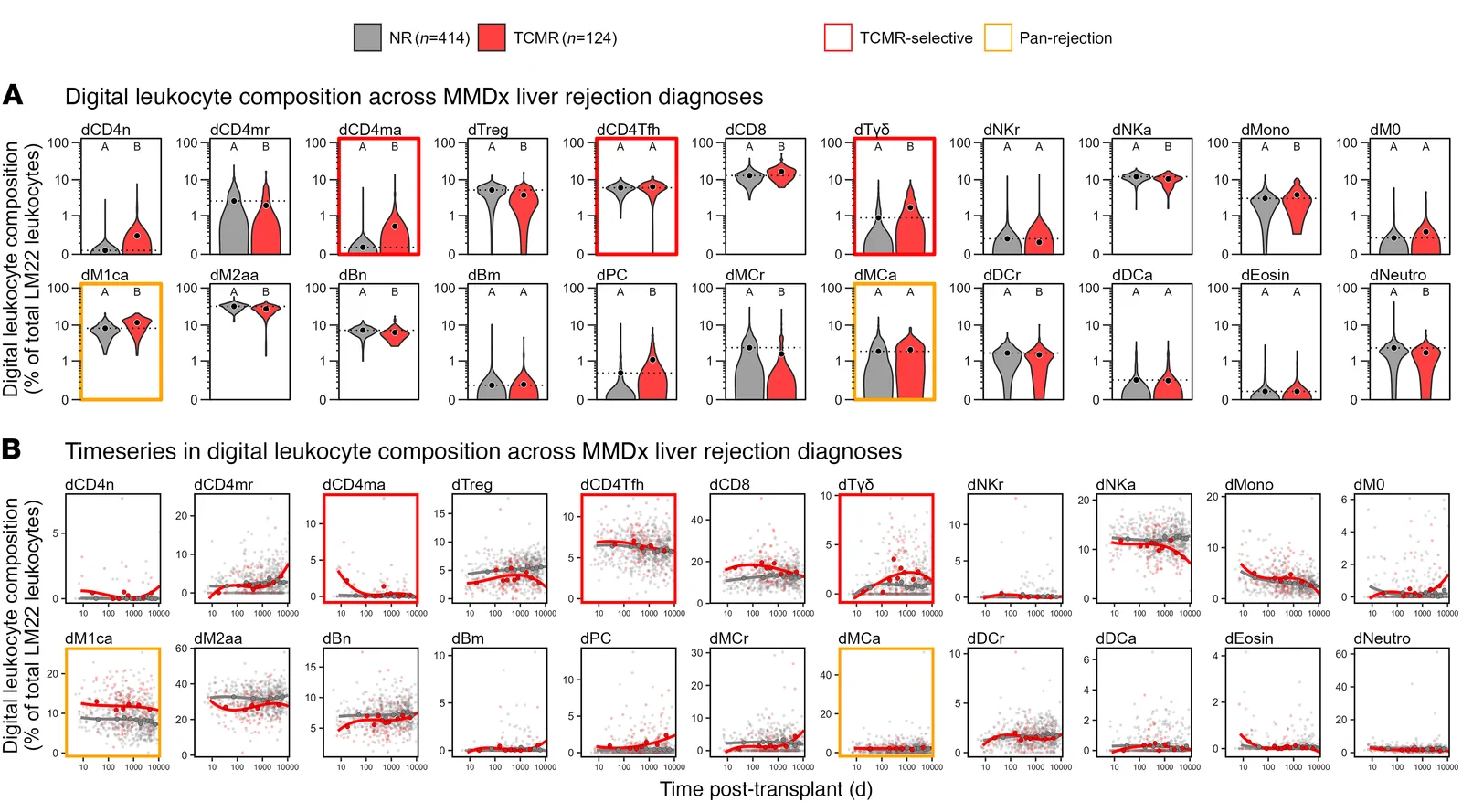
Composition and longitudinal changes in digital leukocytes liver transplant biopsies. Leukocyte profiles were estimated using the CIBERSORTx algorithm and LM22 signature matrix. (**A**) Group-wise comparisons are illustrated as violin plots. Black circles in violin plots depict the mean percent composition by group. Compositional differences in digital leukocytes among rejection groups was analyzed using Bayes moderated *t* tests with arcsin-transformed proportions. Letters at the top of the plot depict a compact letter display of all-pairwise post hoc testing, where groups that share the same letter(s) are not different from one another. (**B**) Time series of estimate leukocytes post-transplant. Small transparent circles depict estimated leukocyte percentages in individual biopsies. Large colored circles depict binned estimated leukocyte percentages using the binscatter method. Curves depict a polynomial fit of estimated leukocyte percentages.

**Figure 7:**
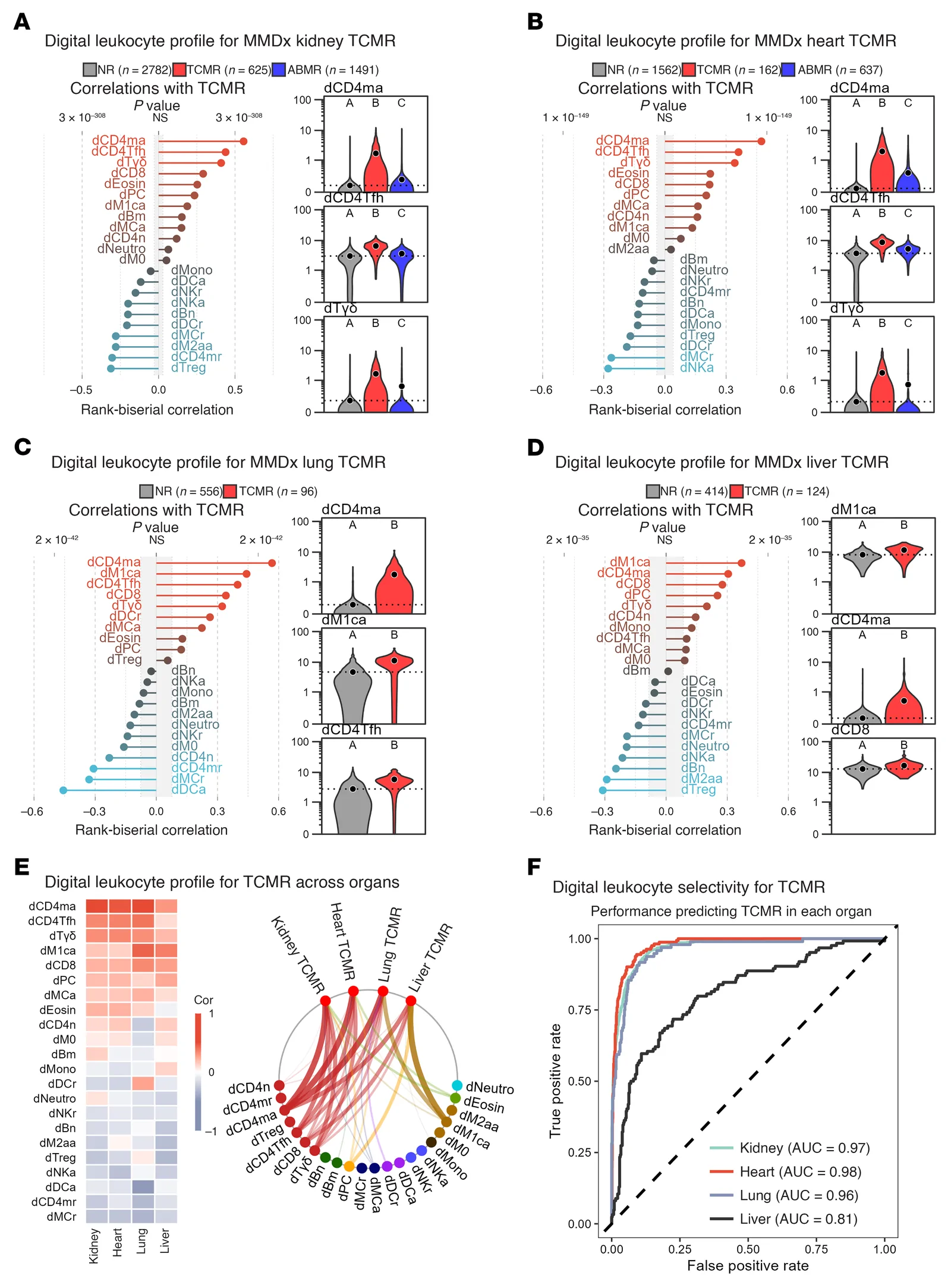
Conserved cross-organ digital leukocyte profile in transplants with T cell–mediated rejection. Digital leukocyte profiles were estimated using the CIBERSORTx algorithm and LM22 signature matrix. (**A**–**D**) Rank biserial correlations (lower *x*-axis) and *P*-values (upper *x*-axis) for 22 leukocytes for T cell–mediated rejection (TCMR) versus all other rejection states in kidney (**A**), heart (**B**), lung (**C**), and liver (**D**) transplant biopsies. Group-wise comparisons of the top 3 positively correlated cell types with TCMR are illustrated as violin plots. Black circles in violin plots depict the mean percent composition by group. Compositional differences in digital leukocytes among rejection groups was analyzed using Bayes moderated *t* tests with arcsin-transformed proportions. Letters at the top of the plot depict a compact letter display of all-pairwise post hoc testing, where groups that share the same letter(s) are not different from one another. (**E**) Clustering leukocyte correlation profiles shows the strong selectivity of CD4ma for TCMR in all organs. A correlation network illustrates positive edges for each cell type with TCMR states in each organ. (**F**) The performance of leukocyte profiles in predicting TCMR was assessed using 10-fold cross-validated generalized linear model with LASSO penalization. Estimated leukocyte profiles performed very well in predicting TCMR in kidney, heart, and lung, but only modestly in liver.

**Figure 8:**
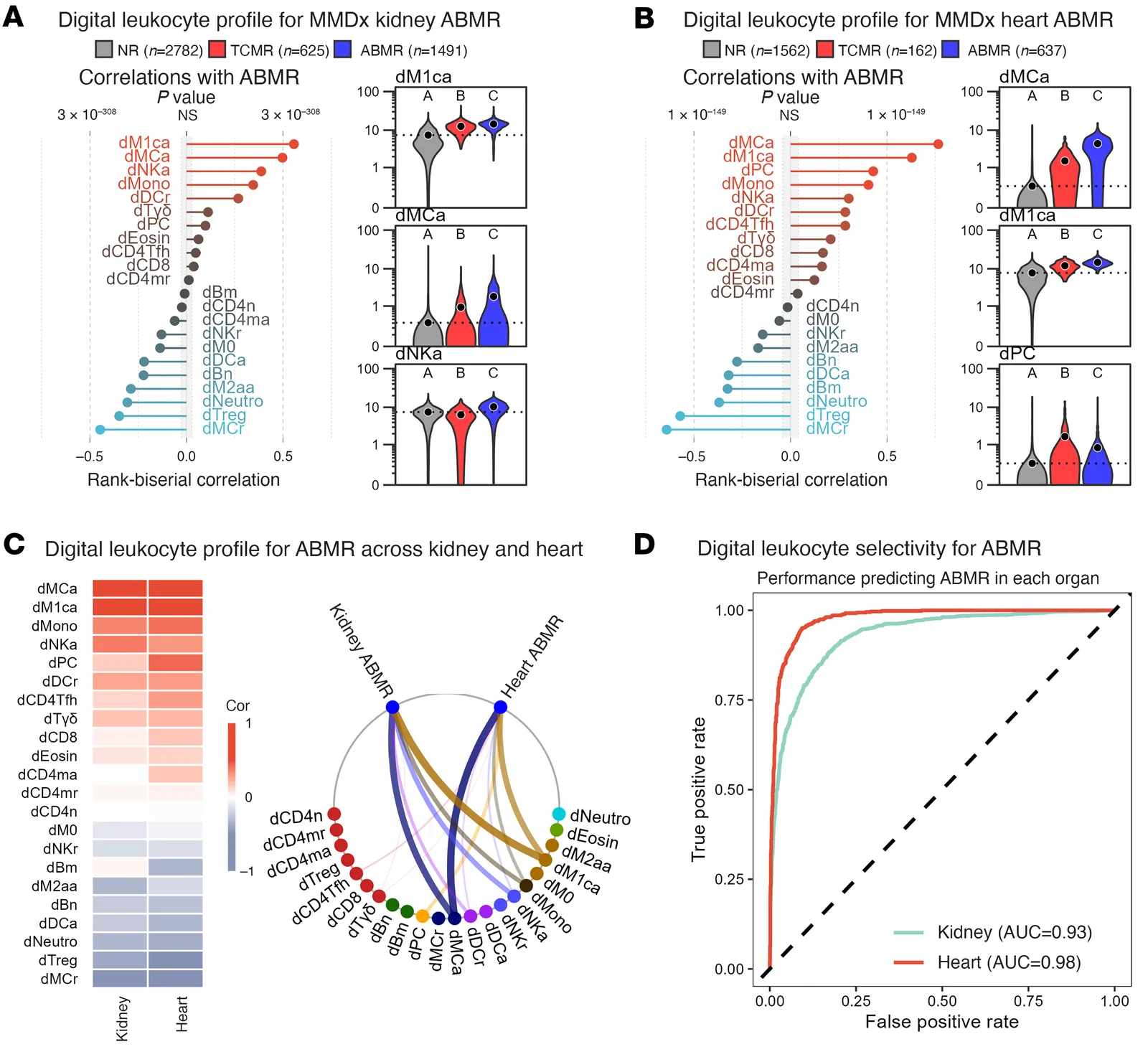
Conserved cross-organ digital leukocyte profile in kidney and heart transplants with antibody-mediated rejection. Digital leukocyte profiles were estimated using the CIBERSORTx algorithm and LM22 signature matrix. (**A** and **B**) Rank biserial correlations (lower *x*-axis) and *P*-values (upper *x*-axis) for 22 leukocytes for antibody-mediated rejection (ABMR) versus all other rejection states in kidney (**A**) and heart (**B**) transplant biopsies. Group-wise comparisons of the top 3 positively correlated leukocyte types with ABMR are illustrated as violin plots. Black circles in violin plots depict the mean percent composition by group. Compositional differences in digital leukocytes among rejection groups was analyzed using Bayes moderated *t* tests with arcsin-transformed proportions. Letters at the top of the plot depict a compact letter display of all-pairwise post hoc testing, where groups that share the same letter(s) are not different from one another. (**C**) Clustering of leukocyte correlations profiles shows the strong selectivity of MCa, M1ca, Mono0, and NKa for ABMR in kidney and heart. A correlation network illustrates positive edges for each leukocyte type with ABMR states in each organ. (**D**) The performance of leukocyte profiles in predicting ABMR was assessed using 10-fold cross-validated generalized linear model with LASSO penalization. Estimated leukocyte profiles performed very well in predicting ABMR in kidney and heart.

**Figure 9:**
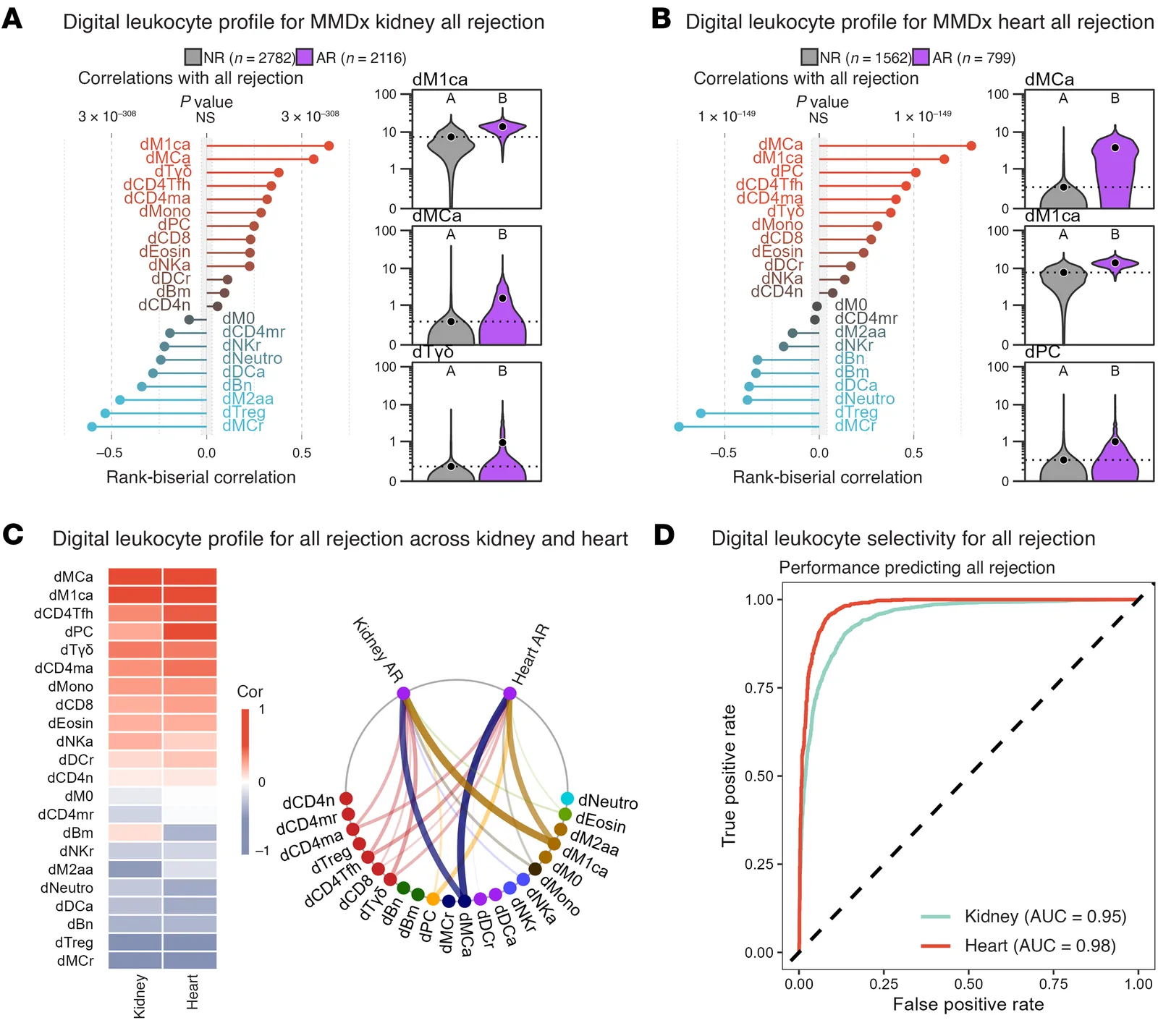
Conserved cross-organ digital leukocyte profile in kidney and heart transplants with all rejection. Leukocyte profiles were estimated using the CIBERSORTx algorithm and LM22 signature matrix. (**A** and **B**) Rank biserial correlations (lower *x*-axis) and *P* values (upper *x*-axis) for 22 leukocytes for no rejection (NR) versus all other rejection states in kidney (**A**) and heart (**B**) transplant biopsies. Group-wise comparisons of the top 3 positively correlated leukocyte types with NR are illustrated as violin plots. Black circles in violin plots depict the mean percentage composition by group. Compositional differences in digital leukocytes among rejection groups was analyzed using Bayes moderated *t* tests with arcsin-transformed proportions. Letters at the top of the plot depict a compact letter display of all-pairwise post hoc testing, where groups that share the same letter(s) are not different from one another. (**C**) Clustering of leukocyte correlation profiles shows the strong selectivity of MCr, Treg, M2aa, and Bn for NR in all organs. A correlation network illustrates positive edges for each leukocyte type with NR states in each organ. (**D**) The performance of leukocyte profiles in predicting NR was assessed using a 10-fold cross-validated generalized linear model with LASSO penalization. Estimated leukocyte profiles performed very well in predicting NR in kidney, heart, and lung, but only modestly in liver. AR, all rejection.

**Figure 10:**
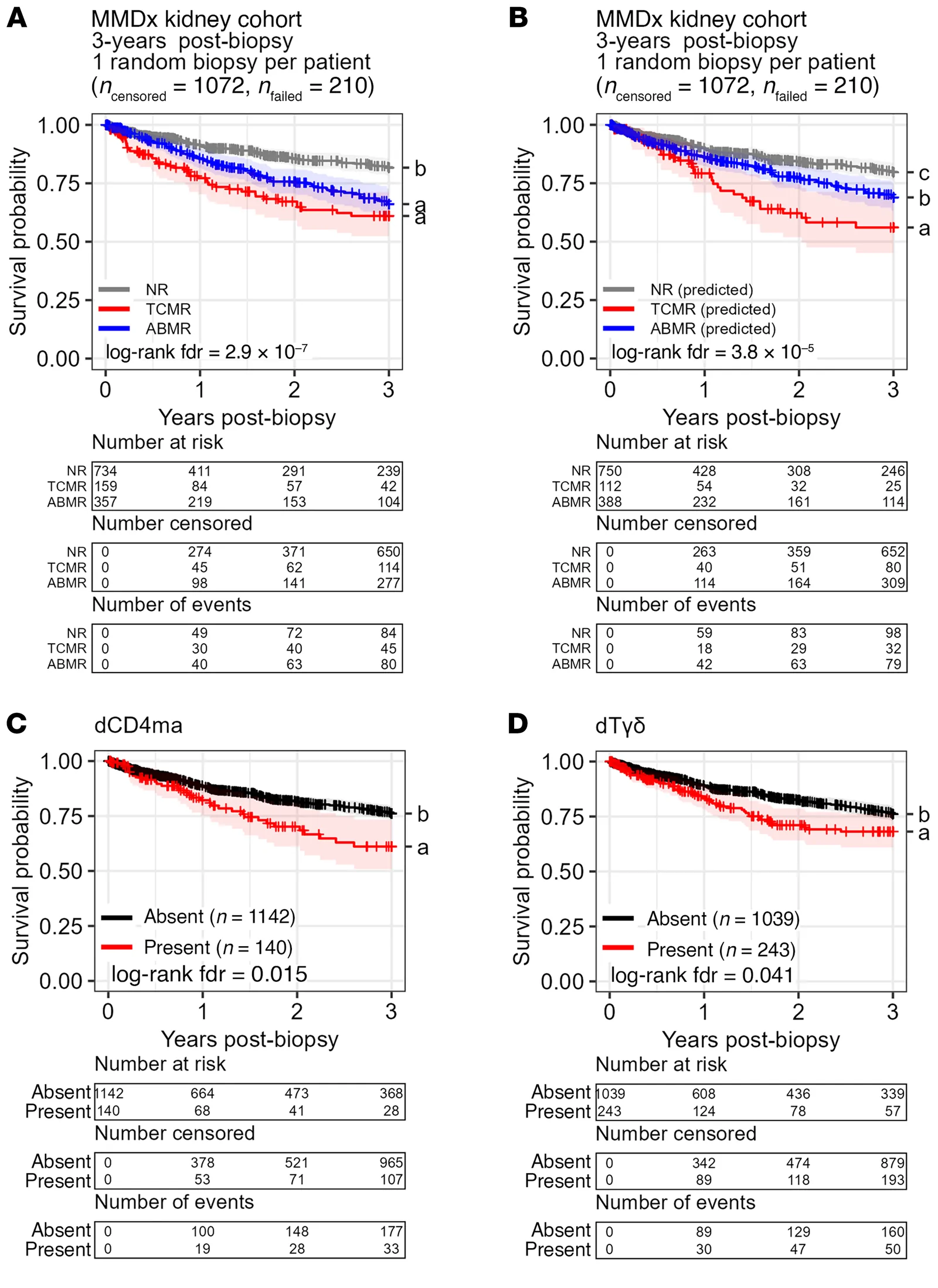
Association of digital leukocytes with graft loss in kidney allografts. Kaplan-Meier curves for kidney allograft survival 3 years after biopsy stratified by MMDx rejection diagnoses (**A**) and predicted molecular rejection diagnoses using cross-validated machine learning trained on MMDx rejection diagnoses using digital leukocyte composition as features (**B**). Kaplan-Meier curves for kidney allograft survival 3 years after biopsy stratified by the presence/absence of (**C**) digital activated memory CD4^+^ T cells (dCD4ma) and (**D**) dTγδ. Overall significance in survival predictions by strata were assessed using a long-rank test. Pairwise differences are represented by a lettering on the right-hand side of each plot, where groups that share the same letter(s) are not different from one another.

**Table 4:**
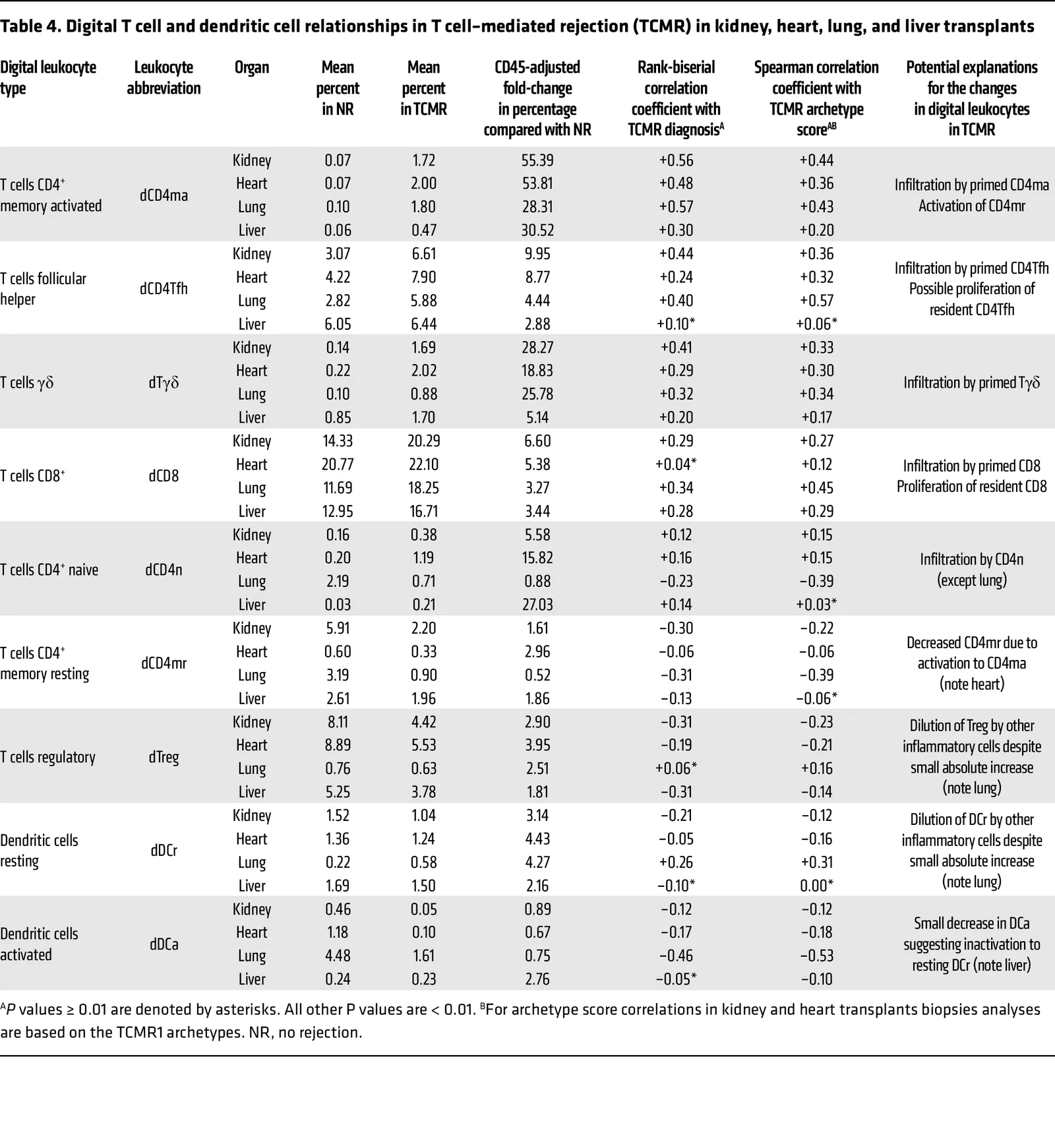
Digital T cell and dendritic cell relationships in T cell–mediated rejection (TCMR) in kidney, heart, lung, and liver transplants

**Table 3:**
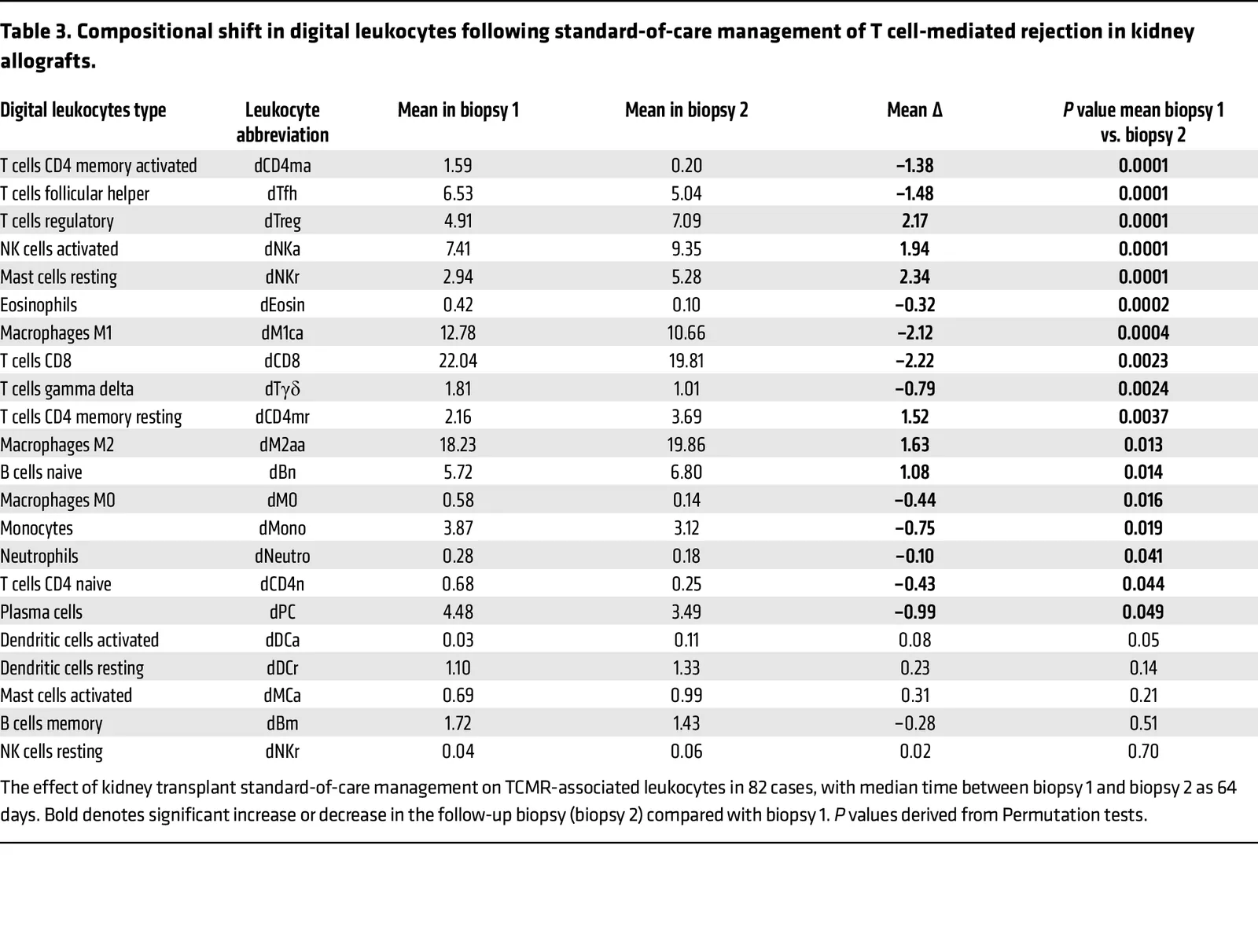
Compositional shift in digital leukocytes following standard-of-care management of T cell-mediated rejection in kidney allografts.

**Table 2:**
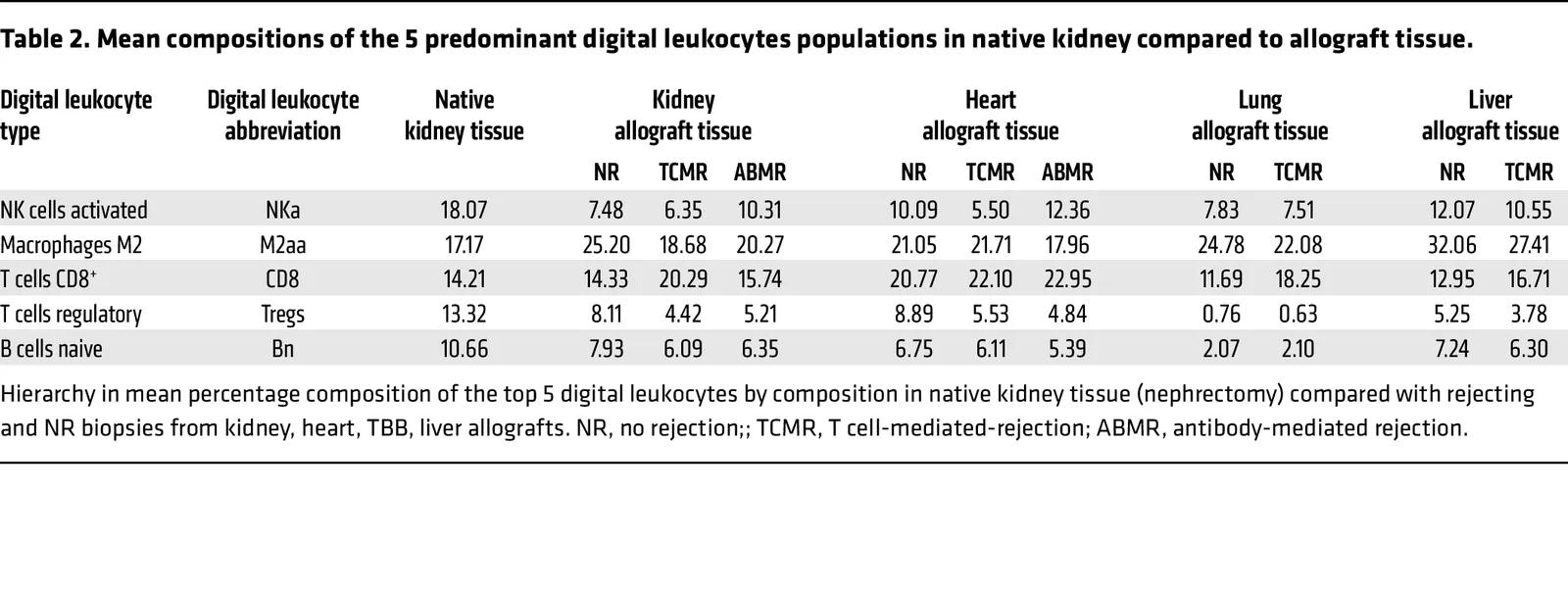
Mean compositions of the 5 predominant digital leukocytes populations in native kidney compared to allograft tissue.

**Table 1:**
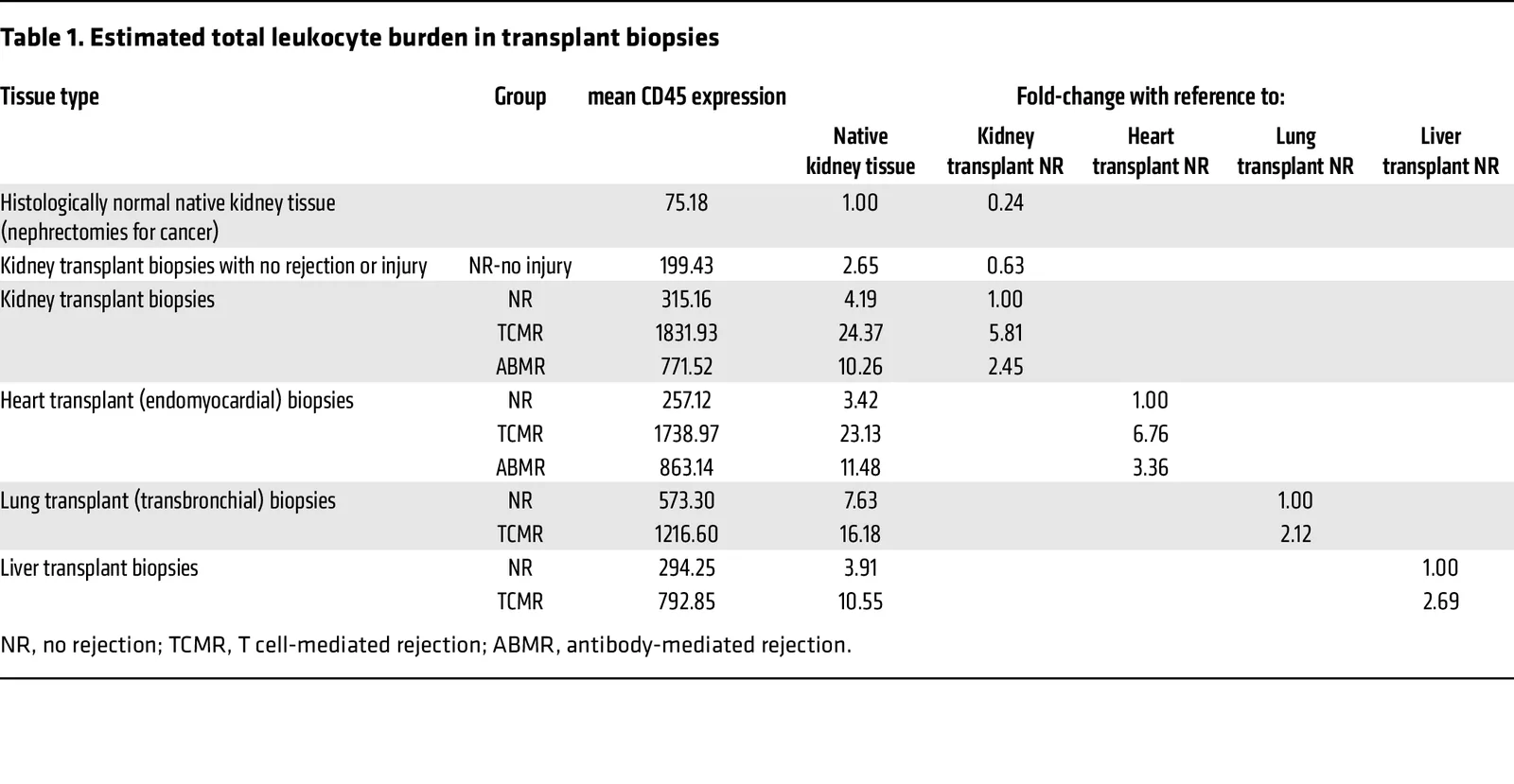
Estimated total leukocyte burden in transplant biopsies

**Table 5:**
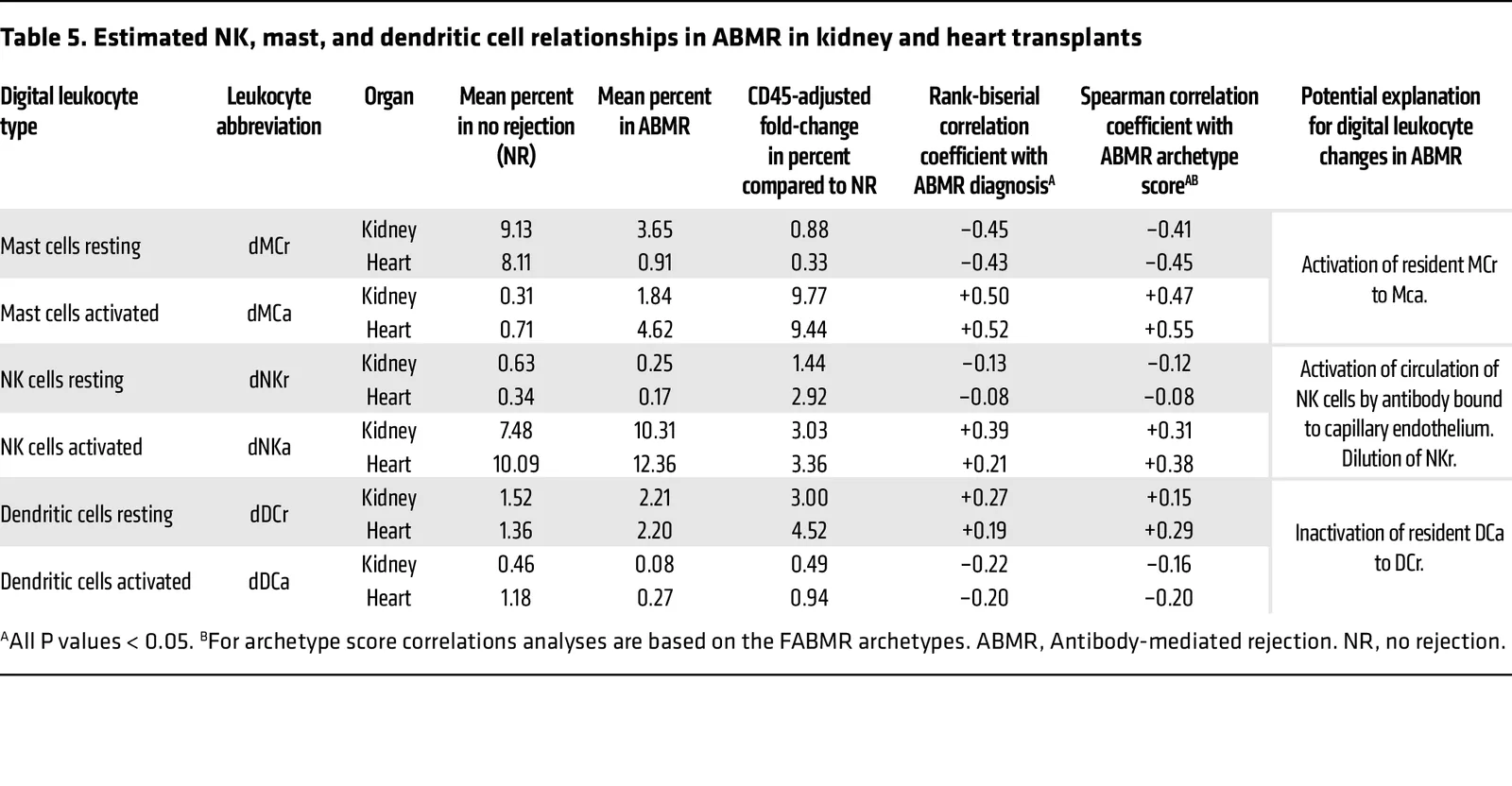
Estimated NK, mast, and dendritic cell relationships in ABMR in kidney and heart transplants

## Version 1 09/22/2026

In-Press Preview

## Version 2 11/16/2026

Electronic publication
