# Supplementary material for: Digital cytometry of leukocyte landscapes reveals shared correlates of rejection across organ transplants
Source: J Clin Invest. 2026 Sep 22;136(22):e208686. doi: 10.1172/JCI208686 (PMC13767621; doi:10.1172/JCI208686)
Supplement: Supplementary file 1 [file jci-136-208686-s287.pdf]

SUPPLEMENTAL MATERIAL

Title

Digital cytometry of leukocyte landscapes reveals shared correlates of rejection across organ transplants

Author list

Patrick T. Gauthier[1,2], Louisa M. S. Gerhardt[3], Lorenz Jahn[4], Christian Hinze[4], Philip F. Halloran*[1,2,5], and the INTERCOMEX, INTERHEART, INTERLUNG, and INTERLIVER Investigators

***Corresponding Author**

Philip F. Halloran, MD, PhD, Alberta Transplant Applied Genomics Centre, #250 Heritage Medical Research Centre, University of Alberta, Edmonton, AB T6G 2S2, Canada.

E-mail: phallora@ualberta.ca

Affiliations

[1]Alberta Transplant Applied Genomics Centre, Edmonton, AB, Canada

[2]Transcriptome Sciences Inc., Edmonton, AB, Canada

[3]Fifth Department of Medicine, Mannheim Medical Faculty of the University of Heidelberg, University Hospital Heidelberg, Mannheim, Germany

[4]Department of Nephrology and Hypertension, Hannover Medical School, Hannover, Germany

[5]University of Alberta, Edmonton, AB, Canada

Table of Contents

Supplemental Results **3**

Digital leukocyte composition in lung transplant transbronchial biopsies. 3

Digital leukocyte composition in liver transplant biopsies. 3

Supplemental Methods **3**

Comparing reference alignment with rejection diagnoses. 3

Signature matrices. 3

Single-nucleus RNA sequencing of kidney allograft biopsies. 4

Kidney allograft scsnRNAseq data. 4

Liver allograft scRNAseq data. 4

Isolation of leukocytes in scsnRNAseq data. 4

Label transfer of LM22 leukocytes in kidney allograft (scsnRNAseq) data. 5

Rejection classification using digital leukocytes. 5

Prognostic associations of digital leukocytes in kidney allograft biopsies. 5

Supplemental Tables **6**

Supplemental Table 1. Reference matrices for digital leukocyte populations. 6

Supplemental Table 2. Details regarding the LM22 signature matrix* 8

Supplemental Table 3. Spearman correlation coefficients (SCC) of % digital leukocyte composition and rejection archetype scores sorted by TCMR1 in kidney 9

Supplemental Table 4. Hierarchy in adjusted fold change (AFC; 95% confidence intervals) sorted by AFC in TCMR in kidney. 10

Supplemental Table 5. Spearman correlation coefficients (SCC) with FABMR archetype scores in kidney and heart sorted by SCC in kidney FABMR. 11

Supplemental Table 6. Hierarchy in adjusted fold change (AFC; 95% confidence intervals) sorted by AFC in ABMR in kidney. 12

Supplemental Table 7. INTERCOMEX, INTERHEART, INTERLIVER, and INTERLUNG Collaborators. 13

Supplemental Table 8. Patient demographics for kidney TCMR samples assessed by single nucleus RNAseq. 15

Supplemental Figures **16**

Supplemental Figure 1. Comparison of reference alignment with rejection diagnoses. 16

Supplemental Figure 2. Digital leukocyte composition in kidney transplant rejection by Banff diagnosis. 17

Supplemental Figure 3 Digital leukocyte composition in heart transplant rejection by ISHLT diagnosis 18

Supplemental Figure 4 Digital leukocyte composition in lung transplant rejection by ISHLTA-grades ... 19

Supplemental Figure 5 Digital leukocyte composition in liver transplant rejection by histology diagnosis. 20

Supplemental Figure 6. Validation of LM22 estimates in kidney and liver transplant bulk transcriptomics data using scsnRNAseq data from kidney and liver allografts. 21

Reference List .... Error! Bookmark not defined.

Supplemental Results

Digital leukocyte composition in lung transplant transbronchial biopsies.

In lung transplant biopsies, TCMR had an increased % of dCD4ma, dCD4Tfh, dTγδ, and dCD8 and reduced % of dCD4mr, as well as an increased % dPC and Eosinophils. Similar to all rejection in kidney and heart, lung TCMR also had increased M1ca and dMCa, with decreased dMCr, suggesting that rejection in lung also converts dMCr to dMCa. Contrary to other organs, Treg composition was not altered by rejection in lungs. However, similar to other organs, dDCa and dNKr in lungs were highest in NR. Some of these changes were affected by time post-transplant. In TCMR, dCD4ma were higher earlier, whereas dCD4Tfh and dTγδ were higher later, suggesting an evolution of the host cognate T cell response.

Digital leukocyte composition in liver transplant biopsies.

In liver transplant biopsies, TCMR had an increased % of dCD4ma, dTγδ, dCD4n, and dCD8, and dPC, and reduced % of dCD4mr. Similar to all rejection in kidney and heart, liver TCMR also had increased M1ca and decreased dMCr, but not increased dMCa. Additionally, contrary to other organs, dCD4Tfh, dM2aa, dDCa, dNKr, and Eosinophils were not altered by rejection in liver. Some of these changes were affected by time post-transplant. In TCMR, dCD4ma were higher earlier, whereas dTγδ were higher later, suggesting an evolution of the host cognate T cell response.

Supplemental Methods

Comparing reference alignment with rejection diagnoses.

Cellular references were assessed for how well their multidimensional structures aligned with MMDx rejection diagnoses. Cell fraction estimates from each reference were ordinated using principal component analysis (PCA). The resulting PCA embedding was used to determine rejection diagnosis cohesion by Silhouette analysis (1). For kidneys and hearts (i.e., three rejection diagnoses), PCs were also associated with phenotypes using Kruskal-Wallis epsilon-squared (eps^2). For lungs and liver (i.e., two rejection diagnoses), rank-biserial correlations (rbcor) were calculated for each PC. We additionally performed permutational analysis of variance (PERMANOVA) to determine the amount of variance explained by rejection diagnosis within each reference PCA embedding. A composite score of weighted averages of Silhouette scores, PERMANOVA r^2, and eps^2/rbcor was included to simplify comparisons of the alignment of each reference with rejection diagnoses. Mean composite scores and 95% confidence intervals were calculated by bootstrap resampling stratified by diagnosis. The composite score ranges from 0 to 1 and demonstrates relative alignment of each reference with diagnosis.

Signature matrices.

The signature matrix for ImmunoStates was obtained from the online supplement associated with the paper (2). The signature matrix (and reference matrix required for S-mode batch correction) for KTB18 was obtained by a request made to the corresponding author of the original publication for KTB18 (3). For HCAv2 (https://www.heartcellatlas.org/), HCLAv1.0 (https://data.humancellatlas.org/hca-bio-networks/lung/atlases/lung-v1-0), and LCA (https://www.livercellatlas.org/), raw scRNAseq count data paired with cell type annotation metadata were obtained and used to produce de novo reference matrices using a maximum of 1,000 cells per cell type to reduce computational load. The CIBERSORTx/fractions module was

used to produce the HCAv2, HCLAv1.0, and LCA signature matrices with the following parameters: single cell = TRUE, g_min = 300, g_max = 5000, q_value = 0.05, filter = FALSE, k_max = 999, replicates = 5, sampling = 0.5, fraction = 0.75 with all other parameters set to default. In cases where genes in the signature matrix were represented by multiple Affymetrix probesets, the probeset with the highest mean expression was used.

Single-nucleus RNA sequencing of kidney allograft biopsies.
A total of 8 samples from 6 patients (Supplemental Table 8) underwent nuclei isolation as previously described (75, 76) and single-cell sequencing using the 10x Genomics Chromium Next GEM Single Cell 3' v3.1 chemistry protocol (#CG000315 Rev E), targeting 10,000 nuclei per sample. Libraries were sequenced on Illumina NovaSeq X Plus (paired-end) and digital expression matrices were generated with the 10x Genomics Cell Ranger software (version 8.0.0).

Kidney allograft scsnRNAseq data.
Additional scsnRNAseq data from kidney allograft biopsies from patients diagnosed with NR, TCMR, and ABMR were gathered from three independent cohorts of kidney transplant biopsies (BioStudies accession code E-MTAB-12051; GEO accession code: GSE195718; GEO accession code: GSE284742), including 24 biopsies from stable/non-rejecting grafts, 3 biopsies from grafts with ABMR, and 4 biopsies from grafts with TCMR (3-5). Raw counts and metadata from our *de novo* dataset and publicly available dataset were processed using the Seurat pipeline in R. Quality control involved filtering cells having fewer than 400 genes (i.e., empty droplets and dead cell filtering), more than 10,001 genes (i.e., doublet filtering), and cells with a mitochondrial gene content > 25% (i.e., mitochondria filtering). This resulted in 167684 cells across 39 samples being retained. Data were integrated using 2000 features using the 'IntegrateData' Seurat workflow, and PCA and UMAP embeddings were calculated.

Liver allograft scRNAseq data.
scRNAseq data from 30 cryopreserved liver biopsies from an independent cohort of liver transplant recipients were re-assessed (GEO accession code: GSE256141), including 6 pre-transplant biopsies, 3 biopsies from stable/non-rejecting grafts, 16 biopsies from grafts with TCMR, and 5 biopsies from grafts with resolved-TCMR post-treatment (6). Raw counts and metadata from the publicly available dataset were processed using the Seurat pipeline in R following parameters used in the original study. Quality control involved filtering out cells having fewer than 250 genes, fewer than 500 unique molecular identifiers, and cells with a mitochondrial gene content > 30%. Pre-transplant, and post-treatment biopsies were excluded, resulting in 10888 cells across 19 samples being retained. Data were then normalized as per standard Seurat workflow, and PCA and UMAP embeddings were calculated.

Isolation of leukocytes in scsnRNAseq data.
Leukocytes were isolated by first clustering cells using the shared nearest neighbors (SNN) and Louvain algorithms using the integrated PCA and UMAP embeddings. Leukocyte clusters were then identified using a mean *PTPRC*/*CD45* expression >1, and for liver scRNAseq data clusters with high expression of Kupffer cell markers (i.e., *MARCO*, *TIMD4*, *VSIG4*, *CD5L*, *C1QA*) were also retained. For the kidney scsnRNAseq cohort this resulted in 27068 leukocytes and 140616 kidney cells. For the liver scRNAseq cohort this resulted in 9902 leukocytes and 986 liver cells. Additional quality control was carried out to exclude NR biopsies with unexplained high leukocyte fractions (i.e., higher than TCMR or ABMR biopsies; >13% leukocyte composition in the kidney cohort), and TCMR or ABMR biopsies with low leukocyte fractions (<13% in

leukocyte composition in the kidney cohort; <maximum leukocyte % composition in NR biopsies). Final analyses included a total of 15 NR, 6 TCMR, and 3 ABMR kidney biopsies, and 3 NR and 14 TCMR liver biopsies.

Label transfer of LM22 leukocytes in kidney allograft (scsnRNAseq) data.
LM22 annotations were imposed on the scsnRNAseq data using the SingleR algorithm (34) using the LM22 source data (https://CIBERSORTxx.stanford.edu/csdownload.php; “LM22 ref (txt)”) and default SingleR settings. Cell types that had low confidence by SingleR prediction scores across all cells were omitted, and SingleR was repeated with the resulting subset of leukocytes.

Rejection classification using digital leukocytes.
To exemplify the consistency in DLE composition with MMDx rejection diagnoses, multiclass generalized linear models with least absolute shrinkage and selection operator (LASSO) for feature reduction (glmnet) were trained to assess the predictive performance of the estimated leukocyte landscape for rejection states in kidney, heart, lung, and liver allografts. Glmnet performances were validated using nested cross-validation with 10 inner folds for hyperparameter tuning, and 10 outer folds for calculating final reported performances and presented as area-under-the-receiver-operator curve (AUC).

Prognostic associations of digital leukocytes in kidney allograft biopsies.
One random biopsy per patient was selected for survival assessments, producing survival cohorts of 1282 kidney (1072 censored, 210 failed). Survival was censored at 3-years post-biopsy. Survival was assessed using Kaplan-Meier estimates (KM) of rejection groups by MMDx diagnoses and labels predicted by the glmnet classifier (described above). KM estimates were also presented for dCD4ma and dTγδ (i.e., the two digital leukocytes most associated with TCMR). dCD4ma and dTγδ were stratified as present/absent (i.e., due to the relative scarcity of these digital leukocytes).

Supplemental Tables

Supplemental Table 1. Reference matrices for digital leukocyte populations.									
**Origin**	**Lineage**	**Cell type**	**Abbreviation**	**LM22**	**ImmunoStates**	**KTB18**	**HCAv2**	**HCLAv1.0**	**LCA**
lymphocyte	T cell	unclassified	T					✓	✓
		CD4	CD4		✓			✓	
		CD4 naive	CD4n	✓		✓	✓		
		CD4 activated	CD4a				✓		
		CD4 memory	CD4m			✓			
		CD4 memory resting	CD4mr	✓					
		CD4 memory activated	CD4ma	✓					
		CD4 regulatory	Tregs	✓			✓		
		CD4 follicular helper	CD4Tfh	✓			✓		
		CD4 helper th1	CD4th1				✓		
		CD4 helper th2	CD4th2				✓		
		CD4 helper th17	CD4th17				✓		
		CD8	CD8	✓	✓			✓	
		CD8 transitioning	CD8trans				✓		
		CD8 effector	CD8e			✓	✓		
		CD8 effector memory	CD8em			✓	✓		
		CD8 cytotoxic	CD8cytox				✓		
		CD8 temra	CD8temra			✓			
		gamma delta	Tγδ	✓	✓		✓		
		MAIT	MAIT				✓		
	NK cell	unclassified	NK					✓	
		activated	NKa	✓					
		resting	NKr	✓					
		CD56low	NKCD56low		✓				
		CD56high	NKCD56high				✓		
		CD16high	NKCD16high				✓		
		CD16high	NKCD16ahigh			✓			
		CD16low	NKCD16alow			✓			
		resident	NKres						✓
	T/NK cell	cycling	T_NKcycling				✓		
		circulating	NK_NKTcirc						✓
	B cell	unclassified	B			✓	✓	✓	✓
		naive	Bn	✓	✓				
		memory	Bm	✓	✓				
		plasmacytoid	Bplas				✓		
	Plasma cell		PC	✓	✓			✓	✓
myeloid cell	Monocyte	Monocyte	Mono0	✓					
		Monocyte	MonoCD16		✓		✓		
		Monocyte	MonoCD16a+			✓			
		Monocyte	MonoMacroCD14+			✓			
		Monocyte	Monoderived						✓
		classical	Mono1c					✓	
		non-classical	Mono2nc					✓	
		CD14	MonoCD14		✓		✓		
	Macrophage	activated	M12					✓	✓
		M0	M0	✓	✓				
		M0	M0alveolar					✓	
		M0	M0lung					✓	
		alternatively-activated	M2aa	✓	✓				
		classically-activated	M1ca	✓	✓				
	Dendritic cell	unclassified	DC				✓	✓	
		activated	DCa	✓					
		resting	DCr	✓					
		plasmacytoid	DCplas		✓			✓	✓
		myeloid	DCmye		✓			✓	
		conventional	DC1c						✓
		conventional	DC2c						✓
		conventional	DCc					✓	
		conventional-migratory	DC2cmig						✓

	Basophil	unclassified	Baso		✓				✓
	Neutrophil	unclassified	Neutro	✓	✓		✓		✓
	Eosinophil	unclassified	Eosin	✓	✓				
	Mast cell	unclassified	MC		✓		✓	✓	
		activated	MCa	✓					
		resting	MCr	✓					
	Progenitor	monocyte–macrophage	MoMP				✓		
		unclassified	Myeloid				✓		
		LYVE1+, cycling	MLYVE1+cycling				✓		
		LYVE1+, IGF1+	MLYVE1+IGF1+				✓		
		LYVE1+, TIMD4+	MLYVE1+TIMD4+				✓		
stem cell	Progenitor	hematopoietic	HPC		✓			✓	

Supplemental Table 2. Details regarding the LM22 signature matrix*

Cell type	Cell subtype	Abbreviation	Reference (PMID)	leukocyte Separation Method	Markers used	Purity
B cells	B cells naive	Bn	15789058	MACS® CD138 microbeads and CD19 microbeads	CD19+CD27- IgG/A-	Not stated
	B cells memory	Bm	15789058	MACS® CD138 microbeads and CD19 microbeads, then FACS	CD19+ CD27+	Not stated
PCs	Plasma cells	PC	15789058	MACS® CD138 microbeads, then FACS	CD20+, CD138+ and CD19+	Not stated
CD8 T	T cells CD8	CD8	15789058	RosetteSep™ CD8+ T-cell enrichment cocktail, CD-8 subset	CD3, CD8, CD45RA	>90
CD4 T cells	T cells CD4 naive	CD4n	16791882	Ficoll, then MACS CD4+ T cell isolation kit	CD4+	>98%
	T cells CD4 memory resting	CD4mr	15789058	Ficoll, then FACS	$CD45RO^{high}$	Not stated
	T cells CD4 memory activated	CD4ma	15789058	Ficoll, then FACS, then activated by anti-CD3 (plate-bound) + anti-CD28 (soluble)	$CD45RO^{high}$; CD69, CD25 for activation	>90%
	T cells follicular helper	CD4Tfh	16791882	Ficoll, then MACS CD4+ T cell isolation kit, then FACS	$CXCR5^{hi}$, $ICOS^{hi}$	>95%
	T cells regulatory	Treg	16702978	Ficoll-Hypaque, then MACS CD4+ T cell isolation kit, then FACS	CD4+ $CD25^{hi}$	>98%
Gamma delta T cells	T cells gamma delta	Tγδ	16339519	Ficoll, then FACS	Not stated	Not stated
NK cells	NK cells resting	NKr	15789058	RosetteSep™ NK-cell enrichment cocktail + CD2 Microbeads	CD56	Not stated
	NK cells activated	NKa	15789058	RosetteSep™ NK-cell enrichment cocktail + CD2 Microbeads + IL2 or IL15 for activation	CD56 + CD69	Not stated
Monocytes and Macrophages	Monocytes	Mono	15789058	MACS® CD14 Microbeads, monocyte subset	N/A	Not stated
	Macrophages M0	M0	15789058	Differentiated from monocytes	None known; identified by morphology and phagocytic capability	Not stated
	Macrophages M1	M1ca	17244792	Histopaque 1.077, then Miltenyi negative selection monocyte isolation kit and LS columns, then differentiated with 1% medium supplement nutridoma-HU + 100 nM M-CSF, then activated with 20 ng/ml IFN-g+ 100 ng/ml LPS	None known; identified by morphology and phagocytic capability	>97% (at monocyte stage)
	Macrophages M2	M2aa	17244792	Histopaque 1.077, then Miltenyi negative selection monocyte isolation kit and LS columns, then differentiated with 1% medium supplement nutridoma-HU + 100 nM M-CSF, then activated with 20 ng/ml IFN-g+ 100 ng/ml LPS and 20 ng/ml IL-4	None known; identified by morphology and phagocytic capability	>97% (at monocyte stage)
Dendritic cells	Dendritic cells resting	DCr	15789058	Monocytes differentiated with 17 ng/ml IL4, and 67 ng/ml GMCSF	N/A	Not stated
	Dendritic cells activated	DCa	15789058	Monocytes differentiated with 17 ng/ml IL4, and 67 ng/ml GMCSF, then stimulated with 1 ug/ml LPS	N/A	Not stated
Mast cells	Mast cells resting	MCr	16339519	Ficoll of cord blood, then 100 ng/ml SCF + 10 ng/ml IL-10 + 5 ng/ml IL-6	N/A	95%
	Mast cells activated	MCa	16339519	Ficoll of cord blood, then 100 ng/ml SCF + 10 ng/ml IL-10 + 5 ng/ml IL-6 + IgE receptor activation	N/A	
Eosinophils	Eosinophils	Eosin	16339519	0.6% Dextran T500, then Percoll gradient (70%, 80%), then negative selection with MACS CD16 Microbeads	N/A	>97%
Neutrophils	Neutrophils	Neutro	16339519, 15789058	0.6% Dextran T500, then Percoll gradient (70%, 80%), then negative selection with MACS anti-CCR3 + anti-mouse IgG Microbeads	CD62L	>97%

*Modified from Newman et al. 2015. Nature Methods 12(5):453-457. PMID: 25822800.

**Supplemental Table 3. Spearman correlation coefficients (SCC) of % digital leukocyte composition and rejection archetype scores sorted by TCMR1 in kidney**											
**Digital leukocyte type**	**Leukocyte abbreviation**	**Kidney (n=4898)**			**Heart (n=2361)**			**TBB (n=652)**		**Liver (n=538)**	
		**NR**	**TCMR1**	**FABMR**	**NR**	**TCMR**	**FABMR**	**NR**	**TCMR**	**NR**	**TCMR**
T cells CD4 memory activated	dCD4ma	-0.33	0.45	0.05	-0.38	0.35	0.02[a]	-0.40	0.51	-0.22	0.24
T cells follicular helper	dCD4Tfh	-0.35	0.37	0.11	-0.49	0.31	0.20	-0.57	0.57	-0.12	0.05[a]
T cells gamma delta	dTγδ	-0.41	0.34	0.18	-0.36	0.28	0.07	-0.33	0.35	-0.20	0.20
T cells CD8	dCD8	-0.24	0.28	0.06	-0.34	0.12	0.08	-0.33	0.47	-0.30	0.34
Eosinophils	dEosin	-0.25	0.23	0.10	-0.21	0.19	0.09	-0.22	0.15	-0.01[a]	-0.03[a]
Macrophages M1	dM1ca	-0.71	0.21	0.47	-0.74	0.16	0.52	-0.31	0.60	-0.43	0.42
T cells CD4 naive	dCD4n	-0.07	0.16	0.01[a]	-0.06	0.14	-0.08	0.42	-0.40	-0.05[a]	0.05[a]
B cells memory	dBm	-0.10	0.13	0.01[a]	0.40	-0.10	-0.28	0.21	-0.04[a]	-0.02[a]	0.03[a]
Mast cells activated	dMCa	-0.58	0.11	0.48	-0.74	0.13	0.59	-0.30	0.28	-0.20	0.10
Macrophages M0	dM0	0.09	0.10	-0.11	0.05	0.09	-0.09	0.25	-0.10	-0.10	0.10
Plasma cells	dPC	-0.27	0.08	0.13	-0.53	0.22	0.33	-0.13	0.27	-0.31	0.32
Neutrophils	dNeutro	0.26	0.00[a]	-0.27	0.39	-0.02[a]	-0.29	-0.04[a]	-0.14	0.20	-0.24
Monocytes	dMono	-0.32	-0.05	0.33	-0.29	-0.16	0.43	0.04[a]	-0.12	-0.15	0.09
Dendritic cells resting	dDCr	-0.11	-0.12	0.15	-0.14	-0.17	0.32	-0.28	0.36	0.09	0.00[a]
Dendritic cells activated	dDCa	0.34	-0.13	-0.15	0.42	-0.20	-0.23	0.52	-0.62	0.16	-0.14
NK cells resting	dNKr	0.29	-0.17	-0.12	0.26	-0.17	-0.08	0.17	-0.36	0.20	-0.19
B cells naïve	dBn	0.37	-0.17	-0.21	0.33	-0.15	-0.14	-0.04[a]	-0.02[a]	0.27	-0.24
Macrophages M2	dM2aa	0.48	-0.22	-0.29	0.14	0.19	-0.25	0.25	0.00[a]	0.32	-0.35
T cells CD4 memory resting	dCD4mr	0.18	-0.23	-0.02[a]	0.05	-0.02[a]	0.09	0.38	-0.40	0.15	-0.09
T cells regulatory	dTreg	0.61	-0.23	-0.36	0.62	-0.22	-0.41	-0.26	0.10	0.28	-0.20
NK cells activated	dNKa	-0.22	-0.24	0.32	-0.16	-0.30	0.39	-0.08	-0.07[a]	0.12	-0.19
Mast cells resting	dMCr	0.65	-0.27	-0.41	0.72	-0.26	-0.47	0.33	-0.40	0.29	-0.19

All *P* values are <0.01 unless otherwise noted.

[a] *P* value ≥ 0.01

Supplemental Table 4. Hierarchy in adjusted fold change (AFC; 95% confidence intervals) sorted by AFC in TCMR in kidney.							
**LM22 leukocyte**	**leukocyte abbreviation**	**Kidney transplants**		**Heart transplants**		**Lung transplants**	**Liver transplants**
		**TCMR**	**ABMR**	**TCMR**	**ABMR**	**TCMR**	**TCMR**
T cells CD4 memory activated	dCD4ma	55.39 (41.94 - 76.46)	3.17 (2.12 - 4.69)	53.81 (36.91 - 81.18)	5.01 (3.35 - 7.54)	28.31 (18.82 - 44.29)	30.52 (10.78 - 92.86)
T cells gamma delta	dTγδ	28.27 (22.63 - 34.95)	5.59 (4.56 - 6.92)	18.83 (14.18 - 24.82)	4.45 (3.15 - 6.13)	25.78 (10.54 - 55.70)	5.14 (3.82 - 6.77)
Eosinophils	dEosin	23.64 (17.42 - 31.81)	3.84 (2.82 - 5.36)	26.32 (13.05 - 52.94)	3.72 (1.98 - 7.10)	7.07 (2.71 - 15.58)	2.81 (1.03 - 5.61)
Plasma cells	dPC	11.72 (10.10 - 13.51)	2.58 (2.22 - 3.01)	8.84 (5.26 - 13.80)	3.89 (2.88 - 5.21)	3.19 (2.20 - 4.39)	6.05 (4.21 - 8.72)
Mast cells activated	dMCa	11.57 (9.16 - 15.01)	9.77 (7.95 - 12.33)	5.60 (4.52 - 6.80)	9.44 (8.31 - 10.80)	2.29 (1.38 - 3.80)	2.96 (2.35 - 3.70)
T cells follicular helper	dCD4Tfh	9.95 (9.28 - 10.70)	2.35 (2.22 - 2.50)	8.77 (7.67 - 10.03)	3.03 (2.74 - 3.32)	4.44 (3.84 - 5.19)	2.88 (2.61 - 3.16)
Macrophages M1	dM1ca	7.19 (6.76 - 7.67)	3.67 (3.48 - 3.87)	6.72 (6.14 - 7.33)	3.51 (3.27 - 3.77)	4.60 (4.00 - 5.29)	3.62 (3.25 - 4.01)
T cells CD8	dCD8	6.60 (6.24 - 6.95)	2.26 (2.16 - 2.37)	5.38 (4.88 - 5.91)	2.83 (2.63 - 3.04)	3.27 (2.80 - 3.80)	3.44 (3.05 - 3.90)
B cells memory	dBm	6.17 (4.94 - 7.69)	1.60 (1.31 - 1.93)	5.87 (1.68 - 11.76)	0.36 (0.04 - 0.86)	1.75 (0.58 - 3.56)	2.32 (0.63 - 7.53)
T cells CD4 naive	dCD4n	5.58 (3.59 - 8.91)	1.12 (0.66 - 1.85)	15.82 (7.78 - 28.91)	0.51 (0.27 - 0.91)	0.88 (0.45 - 1.32)	27.03 (6.46 - 118.34)
Monocytes	dMono	5.20 (4.76 - 5.67)	3.16 (2.94 - 3.39)	5.81 (4.80 - 6.93)	3.92 (3.51 - 4.43)	1.70 (1.32 - 2.20)	3.42 (2.92 - 4.00)
NK cells activated	dNKa	4.15 (3.94 - 4.37)	3.03 (2.92 - 3.15)	2.88 (2.59 - 3.21)	3.36 (3.19 - 3.55)	1.90 (1.72 - 2.11)	2.26 (2.07 - 2.47)
B cells naive	dBn	4.03 (3.69 - 4.41)	1.70 (1.63 - 1.78)	6.03 (4.55 - 7.74)	2.12 (1.95 - 2.31)	2.12 (1.64 - 2.75)	2.31 (2.09 - 2.57)
Neutrophils	dNeutro	3.51 (2.64 - 4.63)	0.82 (0.53 - 1.20)	0.40 (0.16 - 0.80)	1.13 (0.20 - 3.02)	2.21 (1.16 - 3.71)	2.01 (1.70 - 2.39)
Macrophages M2	dM2aa	3.49 (3.33 - 3.67)	1.66 (1.59 - 1.73)	4.89 (4.49 - 5.28)	2.17 (2.01 - 2.34)	1.71 (1.53 - 1.90)	2.22 (2.08 - 2.41)
Dendritic cells resting	dDCr	3.14 (2.81 - 3.47)	3.00 (2.80 - 3.22)	4.43 (3.64 - 5.31)	4.52 (4.04 - 5.08)	4.27 (2.43 - 7.30)	2.16 (1.88 - 2.48)
T cells regulatory	dTreg	2.90 (2.73 - 3.09)	1.41 (1.36 - 1.46)	3.95 (3.48 - 4.45)	1.65 (1.52 - 1.79)	2.51 (1.80 - 3.44)	1.81 (1.60 - 2.03)
Mast cells resting	dMCr	1.91 (1.77 - 2.07)	0.88 (0.83 - 0.94)	1.45 (1.17 - 1.75)	0.33 (0.25 - 0.41)	1.00 (0.84 - 1.14)	1.79 (1.18 - 2.56)
T cells CD4 memory resting	dCD4mr	1.61 (1.35 - 1.89)	1.67 (1.54 - 1.80)	2.96 (1.12 - 5.56)	2.90 (1.98 - 4.03)	0.52 (0.30 - 0.81)	1.86 (1.31 - 2.57)
NK cells resting	dNKr	1.36 (0.83 - 2.01)	1.44 (1.13 - 1.79)	4.25 (1.84 - 7.86)	2.92 (1.24 - 5.87)	1.31 (0.60 - 2.28)	1.00 (0.00 - 3.97)
Macrophages M0	dM0	1.33 (0.95 - 1.80)	0.36 (0.25 - 0.51)	4.29 (2.24 - 7.89)	0.40 (0.20 - 0.74)	1.08 (0.60 - 1.71)	4.42 (2.01 - 9.33)
Dendritic cells activated	dDCa	0.89 (0.63 - 1.18)	0.49 (0.38 - 0.62)	0.67 (0.37 - 1.01)	0.94 (0.73 - 1.17)	0.75 (0.63 - 0.89)	2.76 (1.51 - 4.49)

Supplemental Table 5. Spearman correlation coefficients (SCC) with FABMR archetype scores in kidney and heart sorted by SCC in kidney FABMR.

Digital leukocyte type	Leukocyte abbreviation	Kidney (n=4898)			Heart (n=2361)		
		NR	TCMR1	FABMR	NR	TCMR1	FABMR
Mast cells activated	dMCa	-0.58	0.11	0.48	-0.74	0.13	0.59
Macrophages M1	dM1ca	-0.71	0.21	0.47	-0.74	0.16	0.52
Monocytes	dMono	-0.32	-0.05	0.33	-0.29	-0.16	0.43
NK cells activated	dNKa	-0.22	-0.24	0.32	-0.16	-0.30	0.39
T cells gamma delta	dTγδ	-0.41	0.34	0.18	-0.36	0.28	0.07
Dendritic cells resting	dDCr	-0.11	-0.12	0.15	-0.14	-0.17	0.32
Plasma cells	dPC	-0.27	0.08	0.13	-0.53	0.22	0.33
T cells follicular helper	dCD4Tfh	-0.35	0.37	0.11	-0.49	0.31	0.20
Eosinophils	dEosin	-0.25	0.23	0.10	-0.21	0.19	0.09
T cells CD8	dCD8	-0.24	0.28	0.06	-0.34	0.12	0.08
T cells CD4 memory activated	dCD4ma	-0.33	0.45	0.05	-0.38	0.35	0.02[a]
B cells memory	dBm	-0.10	0.13	0.01[a]	0.40	-0.10	-0.28
T cells CD4 naive	dCD4n	-0.07	0.16	0.01[a]	-0.06	0.14	-0.08
T cells CD4 memory resting	dCD4mr	0.18	-0.23	-0.02[a]	0.05	-0.02	0.09
Macrophages M0	dM0	0.09	0.10	-0.11	0.05	0.09	-0.09
NK cells resting	dNKr	0.29	-0.17	-0.12	0.26	-0.17	-0.08
Dendritic cells activated	dDCa	0.34	-0.13	-0.15	0.42	-0.20	-0.23
B cells naïve	dBn	0.37	-0.17	-0.21	0.33	-0.15	-0.14
Neutrophils	dNeutro	0.26	0.00[a]	-0.27	0.39	-0.02	-0.29
Macrophages M2	dM2aa	0.48	-0.22	-0.29	0.14	0.19	-0.25
T cells regulatory	dTreg	0.61	-0.23	-0.36	0.62	-0.22	-0.41
Mast cells resting	dMCr	0.65	-0.27	-0.41	0.72	-0.26	-0.47

All *P* values are <0.01 unless otherwise noted.

[a] *P* value ≥ 0.01

**Supplemental Table 6. Hierarchy in adjusted fold change (AFC; 95% confidence intervals) sorted by AFC in ABMR in kidney.**					
**leukocyte type**	**leukocyte type abbreviation**	**Kidney transplants**		**Heart transplants**	
		**TCMR**	**ABMR**	**TCMR**	**ABMR**
Mast cells activated	dMCa	11.57 (9.16 - 15.01)	9.77 (7.95 - 12.33)	5.60 (4.52 - 6.80)	9.44 (8.31 - 10.80)
T cells gamma delta	dTγδ	28.27 (22.63 - 34.95)	5.59 (4.56 - 6.92)	18.83 (14.18 - 24.82)	4.45 (3.15 - 6.13)
Eosinophils	dEosin	23.64 (17.42 - 31.81)	3.84 (2.82 - 5.36)	26.32 (13.05 - 52.94)	3.72 (1.98 - 7.10)
Macrophages M1	dM1ca	7.19 (6.76 - 7.67)	3.67 (3.48 - 3.87)	6.72 (6.14 - 7.33)	3.51 (3.27 - 3.77)
T cells CD4 memory activated	dCD4ma	55.39 (41.94 - 76.46)	3.17 (2.12 - 4.69)	53.81 (36.91 - 81.18)	5.01 (3.35 - 7.54)
Monocytes	dMono	5.20 (4.76 - 5.67)	3.16 (2.94 - 3.39)	5.81 (4.80 - 6.93)	3.92 (3.51 - 4.43)
NK cells activated	dNKa	4.15 (3.94 - 4.37)	3.03 (2.92 - 3.15)	2.88 (2.59 - 3.21)	3.36 (3.19 - 3.55)
Dendritic cells resting	dDCr	3.14 (2.81 - 3.47)	3.00 (2.80 - 3.22)	4.43 (3.64 - 5.31)	4.52 (4.04 - 5.08)
Plasma cells	dPC	11.72 (10.10 - 13.51)	2.58 (2.22 - 3.01)	8.84 (5.26 - 13.80)	3.89 (2.88 - 5.21)
T cells follicular helper	dCD4Tfh	9.95 (9.28 - 10.70)	2.35 (2.22 - 2.50)	8.77 (7.67 - 10.03)	3.03 (2.74 - 3.32)
T cells CD8	dCD8	6.60 (6.24 - 6.95)	2.26 (2.16 - 2.37)	5.38 (4.88 - 5.91)	2.83 (2.63 - 3.04)
B cells naive	dBn	4.03 (3.69 - 4.41)	1.70 (1.63 - 1.78)	6.03 (4.55 - 7.74)	2.12 (1.95 - 2.31)
T cells CD4 memory resting	dCD4mr	1.61 (1.35 - 1.89)	1.67 (1.54 - 1.80)	2.96 (1.12 - 5.56)	2.90 (1.98 - 4.03)
Macrophages M2	dM2aa	3.49 (3.33 - 3.67)	1.66 (1.59 - 1.73)	4.89 (4.49 - 5.28)	2.17 (2.01 - 2.34)
B cells memory	dBm	6.17 (4.94 - 7.69)	1.60 (1.31 - 1.93)	5.87 (1.68 - 11.76)	0.36 (0.04 - 0.86)
NK cells resting	dNKr	1.36 (0.83 - 2.01)	1.44 (1.13 - 1.79)	4.25 (1.84 - 7.86)	2.92 (1.24 - 5.87)
T cells regulatory	dTreg	2.90 (2.73 - 3.09)	1.41 (1.36 - 1.46)	3.95 (3.48 - 4.45)	1.65 (1.52 - 1.79)
T cells CD4 naive	dCD4n	5.58 (3.59 - 8.91)	1.12 (0.66 - 1.85)	15.82 (7.78 - 28.91)	0.51 (0.27 - 0.91)
Mast cells resting	dMCr	1.91 (1.77 - 2.07)	0.88 (0.83 - 0.94)	1.45 (1.17 - 1.75)	0.33 (0.25 - 0.41)
Neutrophils	dNeutro	3.51 (2.64 - 4.63)	0.82 (0.53 - 1.20)	0.40 (0.16 - 0.80)	1.13 (0.20 - 3.02)
Dendritic cells activated	dDCa	0.89 (0.63 - 1.18)	0.49 (0.38 - 0.62)	0.67 (0.37 - 1.01)	0.94 (0.73 - 1.17)
Macrophages M0	dM0	1.33 (0.95 - 1.80)	0.36 (0.25 - 0.51)	4.29 (2.24 - 7.89)	0.40 (0.20 - 0.74)

Supplemental Table 7. INTERCOMEX, INTERHEART, INTERLIVER, and INTERLUNG Collaborators.

**INTERCOMEX Collaborators**		
**Name**	**Contributing Institution**	**Location**
Philip F Halloran	Alberta Transplant Applied Genomics Centre	Edmonton, AB, Canada
Klemens Budde	Charite-Medical University of Berlin	Berlin, Germany
Richard Fatica, Emilio Poggio & Ziad Zaky	Cleveland Clinic Foundation	Cleveland, OH, USA
Bertram Kasiske	Hennepin County Medical Centre	Minneapolis, MN, USA
Iman Francis & Anita Patel	Henry Ford Transplant Institute	Detroit, MI, USA
Ondrej Viklicky, Petra Hruba, Silvie Rajnochová Bloudíčkova & Janka Slatinská	Institute for Experimental and Clinical Medicine	Prague, Czech Republic
Sanjiv Anand	Intermountain Transplant Services	Murray, UT, USA
Daniel Brennan, Sam Kant & Ashish Solanki	Johns Hopkins University School of Medicine	Baltimore, MD, USA
Declan de Freitas & Michael Picton	Manchester Royal Infirmary	Manchester, UK
Justyna Fryc & Beata Naumnik	Medical University in Bialystok	Białystok, Poland
Andrzej Chamienia, Alicja Debska-Ślizień & Joanna Konopa	Medical University of Gdańsk	Gdańsk. Poland
Gunilla Einecke	Medical University of Hannover	Hannover, Germany
Georg Böhmig & Farsad Eskandary	Medical University of Vienna	Vienna, Austria
Mirosław Banasik	Medical University of Wrocław	Wrocław. Poland
Adam Bingaman	Methodist Transplant and Specialty Hospital	San Antonio, TX, USA
Enver Akalin	Montefiore Medical Center	Bronx, NY, USA
Seth Narins & Harold Yang	PinnacleHealth Transplant Associates	Harrisburg, PA, USA
Marek Myślak, Leszek Domański, Marta Gryczman & Joanna Mazurkiewicz	Pomeranian Medical University	Szczecin, Poland
Grzegorz Piecha & Andrzej Wiecek	Silesian Medical University	Katowice, Poland
John Gill, Jagbir Gill & James Lan	St. Paul's Hospital	Vancouver, BC, Canada
Rajendra Baliga & Mahmoud Kamel	Tampa General Hospital	Tampa, FL, USA
Ryszard Grenda	The Children's Memorial Health Institute	Warsaw, Poland
Kevin Chow & Peter Hughes	The Royal Melbourne Hospital	Parkville, Australia
Arskarapurk Jittirat	University Hospital Cleveland Medical Center	Cleveland, OH, USA
Zeljka Jurekovic & Ksenija Vucur	University Hospital Merkur	Zagreb, Croatia
Zbigniew Włodarczyk	University Hospital no. 1	Bydgoszcz, Poland
Thomas Mueller & Thomas Schachtner	University Hospital Zurich	Zurich, Switzerland
Roslyn Mannon	University of Alabama at Birmingham	Birmingham, AL, USA
Sita Gourishankar, Soroush Shojai & Majid Sikosana	University of Alberta	Edmonton, AB, Canada
Miha Arnol, Nika Kojc & Željka Veceric Haler	University of Ljubljana	Ljubljana, Slovenia
Jonathan Bromberg & Matthew R. Weir	University of Maryland	Baltimore, MD, USA
Milagros Samaniego-Picota	Henry Ford Health	Detroit, MI, USA
Sung Shin	University of Ulsan College of Medicine/Assan Medical Center	Seoul, South Korea
Chris Blosser	University of Washington	Seattle, WA, USA
Arjang Djamali	University of Wisconsin	Madison, WI, USA
Arthur Matas	University on Minnesota	Minneapolis, MN, USA
Marius Miglinas	Vilnius University Hospital Santaros Klinikos	Vilnius, Lithuania
Gaurav Gupta & Layla Kamal	Virginia Commonwealth University	Richmond, VA, USA
Michał Ciszek, Dominika Dęborska-Materkowska, Magdalena Durlik & Agnieszka Perkowska-Ptasińska	Warsaw Medical University	Warsaw, Poland
Tarek Alhamad	Washington University at St. Louis	St. Louis, MO, USA
Maciej Glyda	Wojewodzki Hospital	Poznan, Poland
**INTERHEART Collaborators**		
**Name**	**Contributing Institution**	**Location**
Daniel Kim	University of Alberta	Edmonton, AB, Canada
Jon Kobashigawa	Cedars-Sinai Medical Center	Los Angeles, CA, USA
Josef Stehlik	University of Utah	Salt Lake City, UT, USA
Mario Deng	University of California Los Angeles	Los Angeles, CA, USA
Martin Cadeiras	University of California Davis	Davis, CA, USA
Eugene Depasquale	University of Southern California	Los Angeles, CA, USA
Keyur Shah	Virginia Commonwealth University	Richmond, VA, USA
Luciano Potena	University of Bologna	Bologna, Italy
Marisa Crespo	A Coruña University Hospital	A Coruña, Spain
Andreas Zuckermann, Arezu Zuckermann-Aliabadi & Roxana Moayedifar	Medical University of Vienna	Vienna, Austria
Peter Macdonald	St. Vincent's Hospital	Sydney, Australia
Shelley Hall, Joost Felius & Neville Maliakkal	Baylor, Scott & White Health	Dallax, TX, USA
Vojtech Melenovsky	Institute for Experimental and Clinical Medicine (IKEM)	Prague, Czech Republic
**INTERLIVER Collaborators**		
**Name**	**Contributing Institution**	**Location**
Michal Ciszek, Magdalena Durlik, Bartosz Foroncewicz, Michal Grat, Krzysztof Mucha,	Warsaw Medical University	Warsaw, Poland

Maciej Krasnodebski, Agnieszka Perkowska-Ptasinska, and Olga Tronina		
Marwan Abouljoud, Iman Francis, and Dilip Moonka	Henry Ford Hospital	Detroit, MI, USA
Marta Gryczman, Krzysztof Jurczyk, Joanna Mazurkiewicz, Marek Myślak, Marta Wawrzynowicz-Syczewska, and Samir Zeair	Pomeranian Medical University	Szczecin, Poland
Grzegorz Piecha	Medical University of Silesia	Katowice, Poland
Chandra Bhati, Marlon Levy, Trevor Reichman, and Amit Sharma	Virginia Commonwealth University	Richmond, VA, USA
Sandy Feng	University of California San Francisco	San Francisco, CA, USA
David Bruno, Stephen Gray, and Daniel Maluf	University of Maryland	Baltimore, MD, USA
Geoff McCaughan	University of Sydney	Sydney, NSW, Australia
Aldo Montano-Loza	University of Alberta	Edmonton, AB, Canada
Alberto Sanchez-Fueyo	King's College London	London, UK
Catherine Kling, Martin Montenovo, and Jorge Reyes	University of Washington	Seattle, WA, USA
Amar Gupta and Goran Klintmalm	Baylor University Medical Center	Dallas, TX, USA
Seth Karp and Roman Perri	Vanderbilt University	Nashville, TN, USA
**INTERLUNG Collaborators**		
**Name**	**Contributing Institution**	**Location**
Irina Timofte [A]	University of Maryland	Baltimore, MD, USA
Kieran Halloran, Alim Hirji & Justin Weinkauf	University of Alberta	Edmonton, AB, Canada
Greg I Snell & Glen P Westall	Alfred Hospital Lung Transplant Service	Melbourne, Australia
Jan Havlin, Robert Lischke, Andrea Zajacova & Jan Simonek	University Hospital Motol	Prague, Czech Republic
Debbie Levine [B]	University of Texas San Antonio	San Antonio, TX, USA
Ramsey Hachem [C] & Daniel Kreisel	Washington University in St. Louis	St. Louis, MO, USA
Bartosz Kubisa [D] & Maria Piotrowska	Pomeranian Medical University of Szczecin	Szczecin, Poland
Stephen Juvet & Shaf Keshavjee	Toronto Lung Transplant Program – University Health Network	Toronto, ON, Canada
Peter Jaksch	Medical University of Vienna	Vienna, Austria

[A] Now at UT Southwestern
[B] Now at Stanford
[C] Now at University of Utah
[D] Now at Warsaw Medical University

Supplemental Table 8. Patient demographics for kidney TCMR samples assessed by single nucleus RNAseq.	
	**TCMR**<br>(N=6)
**Recipient sex**	
Male	3 (50.0%)
Female	3 (50.0%)
**Recipient age (years)**	68.4 (65.4, 71.5)
**Donor sex**	
Male	0 (0%)
Female	6 (100%)
**Donor age (years)**	70.0 (66.0, 77.0)
**Donor serum creatinine (umol/L)**	62.0 (45.0, 67.0)
Missing	1 (16.7%)
**Critical ischemia time (hours)**	11.5 (2.4, 15.6)
**Induction therapy**	
Anti-IL-2 antibody	6 (100%)
**Immunosuppression**	
Cyclosporin A - Mycophenolate Mofetil - Steroid	6 (100%)
**eGFR at 1 year post KT (ml/min/1.73m2)**	56.8 (39.3, 61.5)

Supplemental Figures

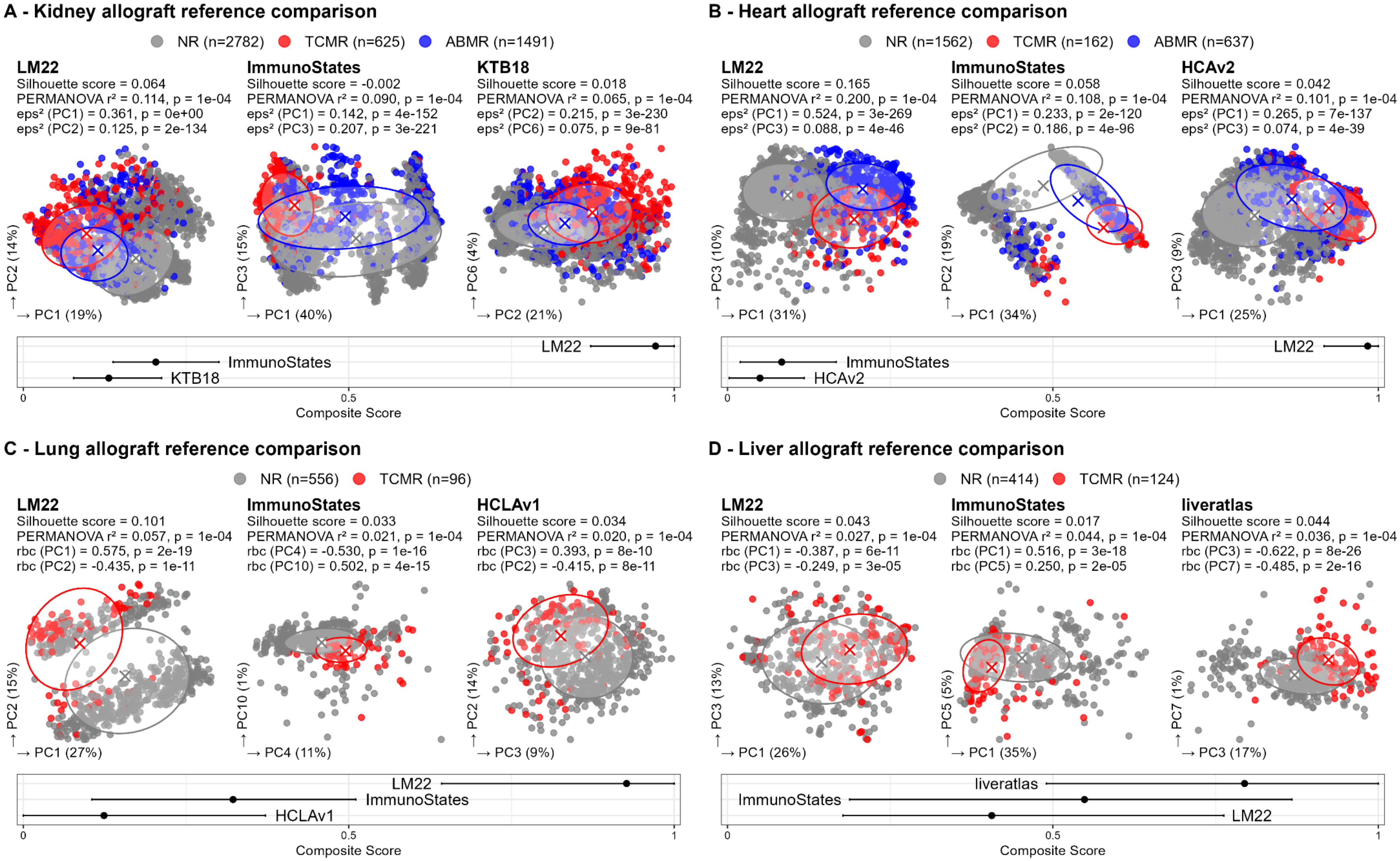

Supplemental Figure 1. Comparison of reference alignment with rejection diagnoses. Cell fraction estimates were ordinated using principal component analysis (PCA) and then compared for their separation of existing rejection diagnoses in kidney **(A)**, heart **(B)**, lung **(C)**, and liver **(D)** allografts. Rejection diagnosis associations were assessed with Silhouette scores using all PCs. Permutational multivariate analysis of variance (PERMANOVA) was used to calculate r^2 describing overall variability in cell fraction estimates with rejection diagnoses. All PCs were assessed for their associations with rejection diagnoses using Kruskal-Wallace epsilon-squared (eps2) for kidney and heart (i.e., >2 groups), and rank biserial correlations (rbc) for lung and liver (i.e., <3 groups). A composite score was then calculated by ranking and weighting Silhouette scores, PERMANOVA r^2, and eps^2/rbc. The composite scores represent the proportion of highest summed ranks across 1000 bootstrapped resamples. Error bars on composite scores denote 95% confidence intervals.

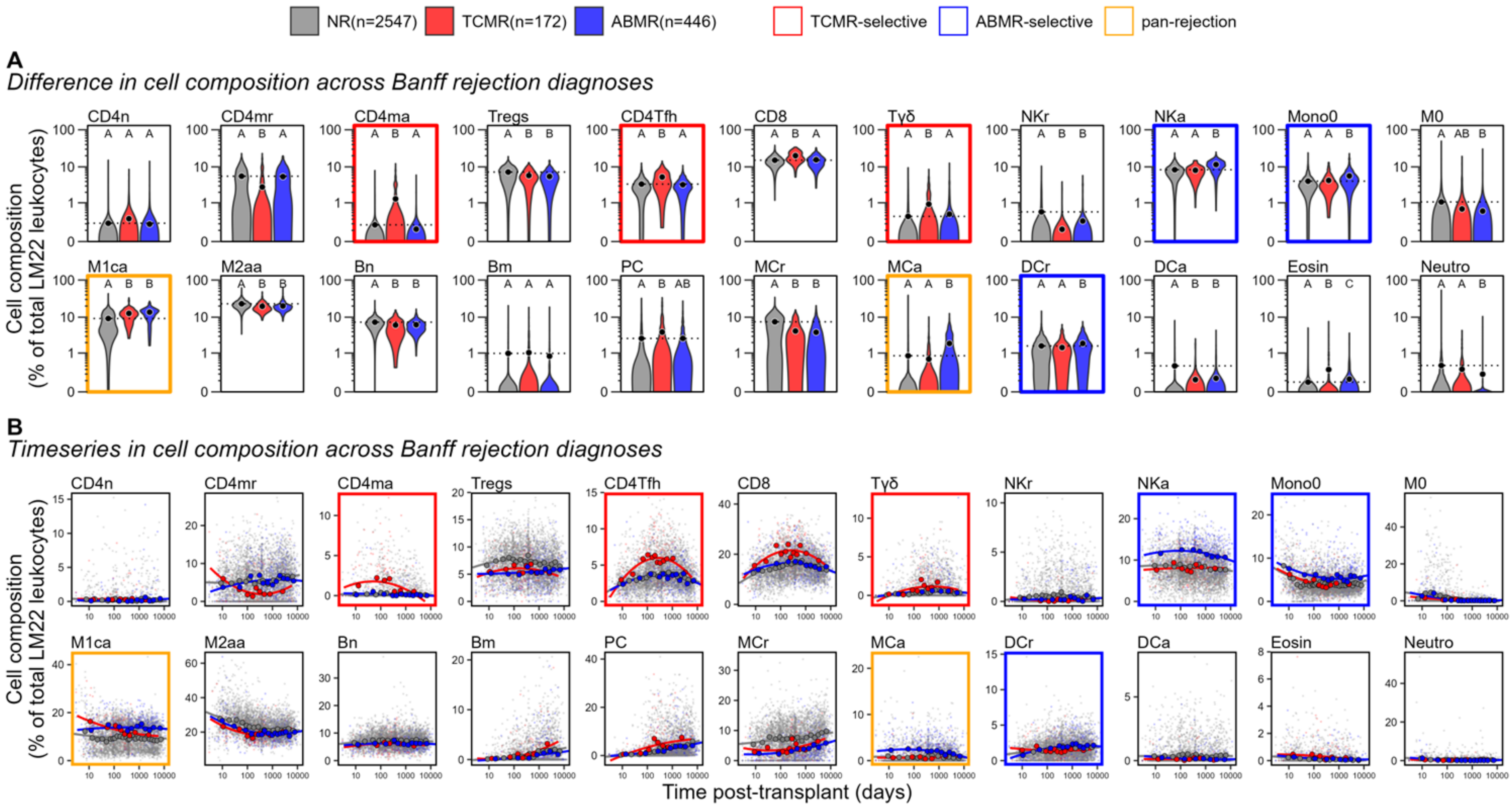

Supplemental Figure 2. Digital leukocyte composition in kidney transplant rejection by Banff diagnosis. Digital leukocyte profiles were estimated using the CIBERSORTx algorithm and LM22 signature matrix. **(A)** Group-wise comparisons are illustrated as violin plots. Black circles in violin plots depict the mean percent composition by group. Compositional differences in digital leukocytes among rejection groups was analyzed using Bayes moderated t-tests with arcsin-transformed proportions. Letters at the top of the plot depict a compact letter display of all-pairwise post-hoc testing, where groups that share the same letter(s) are not different from one another. **(B)** Timeseries of digital leukocytes post-transplant. Small transparent circles depict estimated leukocyte percentages in individual biopsies. Large colored circles depict binned estimated leukocyte percentages using the binscatter method. Curves depict a polynomial fit of estimated leukocyte percentages.

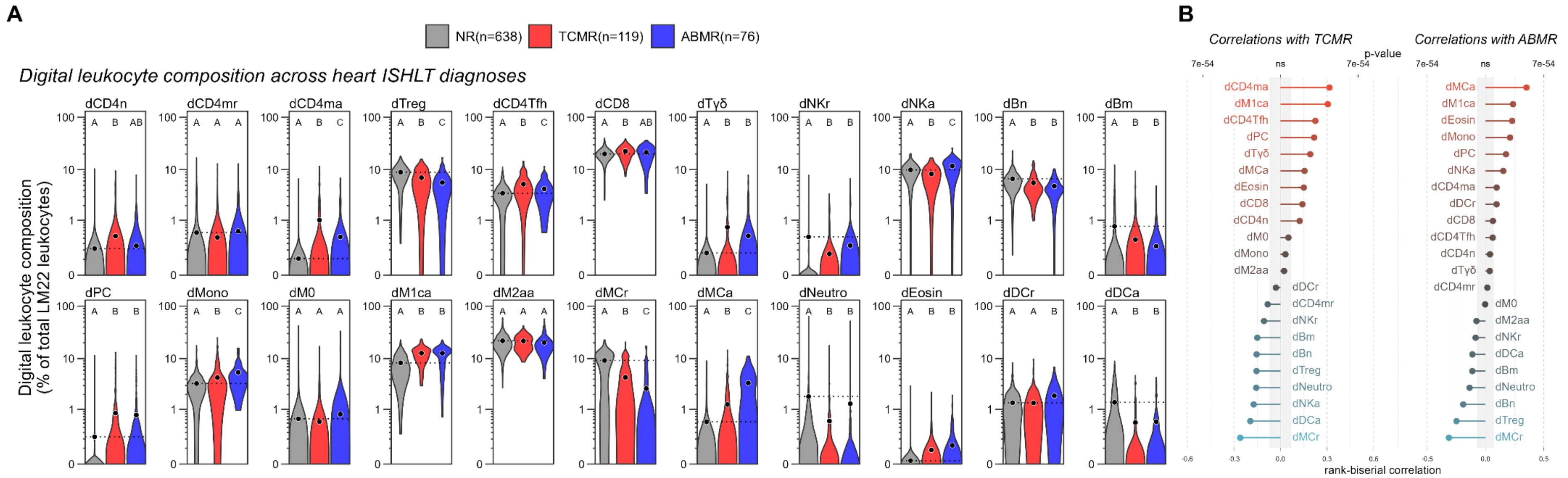

Supplemental Figure 3 Digital leukocyte composition in heart transplant rejection by ISHLT diagnosis. Digital leukocyte profiles were estimated using the CIBERSORTx algorithm and LM22 signature matrix. **(A)** Group-wise comparisons are illustrated as violin plots. Black circles in violin plots depict the mean percent composition by group. Compositional differences in digital leukocytes among rejection groups was analyzed using Bayes moderated t-tests with arcsin-transformed proportions. Letters at the top of the plot depict a compact letter display of all-pairwise post-hoc testing, where groups that share the same letter(s) are not different from one another. **(B)** Rank biserial correlations (lower x-axis) and p-values (upper x-axis) for 22 leukocytes for TCMR versus all other rejection states and ABMR vs all other rejection states in heart.

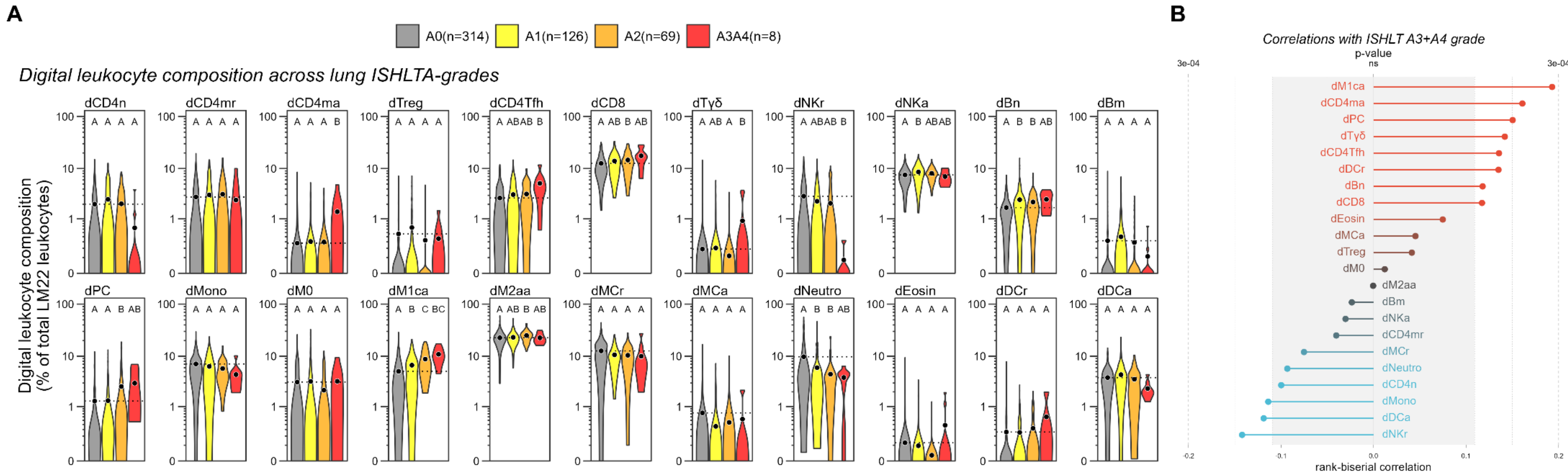

Supplemental Figure 4 Digital leukocyte composition in lung transplant rejection by ISHLTA-grades. Digital leukocyte profiles were estimated using the CIBERSORTx algorithm and LM22 signature matrix. **(A)** Group-wise comparisons are illustrated as violin plots. Black circles in violin plots depict the mean percent composition by group. Compositional differences in digital leukocytes among rejection groups was analyzed using Bayes moderated t-tests with arcsin-transformed proportions. Letters at the top of the plot depict a compact letter display of all-pairwise post-hoc testing, where groups that share the same letter(s) are not different from one another. **(B)** Rank biserial correlations (lower x-axis) and p-values (upper x-axis) for 22 leukocytes for ISHLT A3+A4 versus ISHLT A0 in lung.

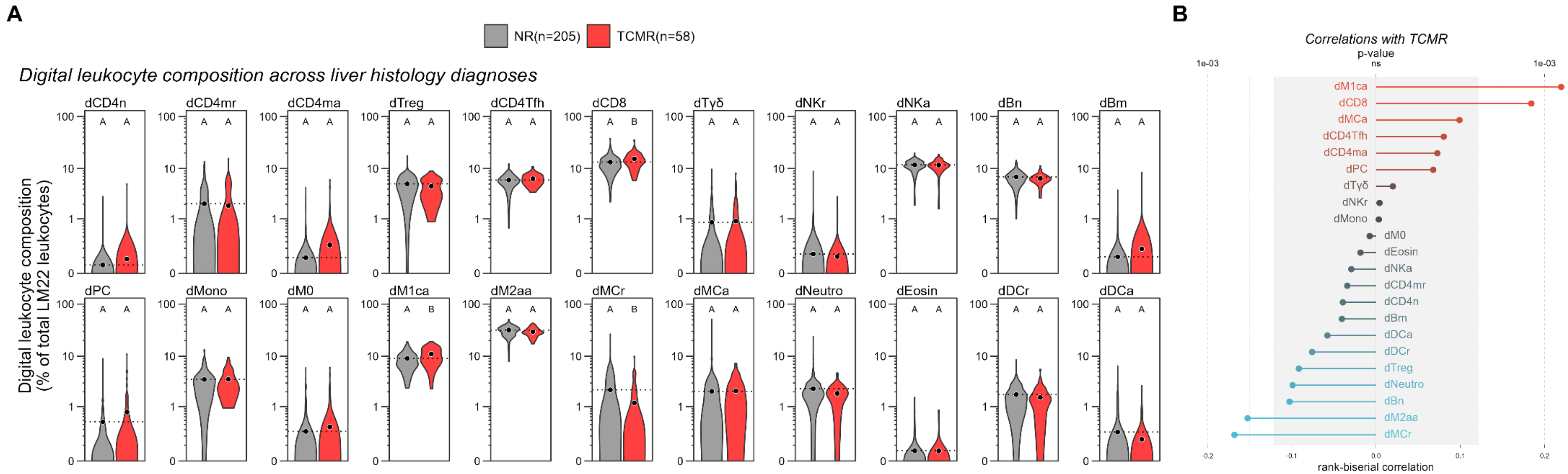

Supplemental Figure 5 Digital leukocyte composition in liver transplant rejection by histology diagnosis. Digital leukocyte profiles were estimated using the CIBERSORTx algorithm and LM22 signature matrix. **(A)** Group-wise comparisons are illustrated as violin plots. Black circles in violin plots depict the mean percent composition by group. Compositional differences in digital leukocytes among rejection groups was analyzed using Bayes moderated t-tests with arcsin-transformed proportions. Letters at the top of the plot depict a compact letter display of all-pairwise post-hoc testing, where groups that share the same letter(s) are not different from one another. **(B)** Rank biserial correlations (lower x-axis) and p-values (upper x-axis) for 22 leukocytes for TCMR versus NR in liver.

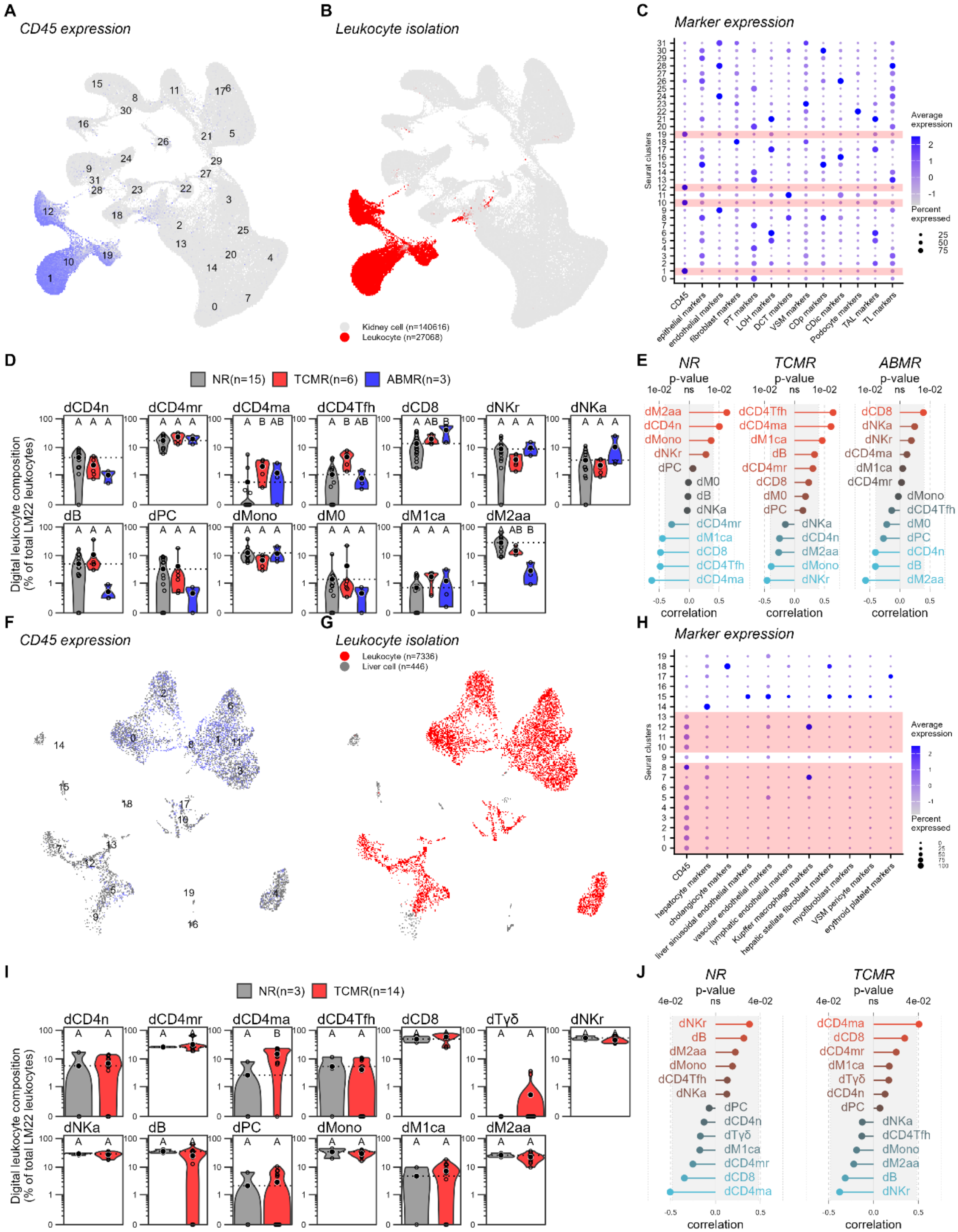

Supplemental Figure 6. Validation of LM22 estimates in kidney and liver transplant bulk transcriptomics data using scsnRNAseq data from kidney and liver allografts. (**A**) CD45 expression and Seurat clustering from kidney scsnRNAseq data. (**B**) Leukocyte isolation from kidney scsnRNAseq data. (**C**) Marker module expression from kidney scsnRNAseq data. (**D**) Violin plots depicting group-wise comparisons of LM22 leukocytes. (**E**) Rank biserial correlations (lower x-axis) and p-values (upper x-axis) for LM22 leukocytes for each rejection group vs all others. (**F**) CD45 expression and Seurat clustering from liver scRNAseq data. (**G**) Leukocyte isolation from liver scRNAseq data. (**H**) Marker module expression from liver scRNAseq data. (**I**) Violin plots depicting group-wise comparisons of LM22 leukocytes. (**J**) Rank biserial correlations (lower x-axis) and p-values (upper x-axis) for LM22 leukocytes for each rejection group vs all others. Empty black circles in panels **D** and **I** depict individual biopsy results. Solid black circles in panels b and e depict the mean percent composition by group. Letters at the top of each plot in panels **D** and **I** depict a compact letter display of all-pairwise post-hoc testing, where groups that share the same letter(s) are not different from one another.

Reference List

1. Rousseeuw PJ. Silhouettes - a Graphical Aid to the Interpretation and Validation of Cluster-Analysis. *Journal of Computational and Applied Mathematics.* 1987;20:53-65.
2. Vallania F, Tam A, Lofgren S, Schaffert S, Azad TD, Bongen E, et al. Leveraging heterogeneity across multiple datasets increases cell-mixture deconvolution accuracy and reduces biological and technical biases. *Nat Commun.* 2018;9(1):4735.
3. Lamarthee B, Callemeyn J, Van Herck Y, Antoranz A, Anglicheau D, Boada P, et al. Transcriptional and spatial profiling of the kidney allograft unravels a central role for FcyRIII+ innate immune cells in rejection. *Nat Commun.* 2023;14(1):4359.
4. McDaniels JM, Shetty AC, Kuscu C, Kuscu C, Bardhi E, Rousselle T, et al. Single nuclei transcriptomics delineates complex immune and kidney cell interactions contributing to kidney allograft fibrosis. *Kidney Int.* 2023;103(6):1077-92.
5. Pfefferkorn AM, Jahn L, Gauthier PT, Kulow VA, Roeles J, Muller-Botticher N, et al. Injured epithelial cell states impact kidney allograft survival after T-cell-mediated rejection. *Nat Commun.* 2026;17(1):1060.
6. Peters AL, DePasquale EAK, Begum G, Roskin KM, Kotliar M, Barski A, et al. Single-cell transcriptional landscape of liver transplant rejection reveals tissue persistence of clonally expanded, treatment-resistant T cells. *Am J Transplant.* 2025;25(11):2345-60.
